# Prognostic cancer gene signatures share common regulatory motifs

**DOI:** 10.1038/s41598-017-05035-3

**Published:** 2017-07-06

**Authors:** Ying Wang, Steve Goodison, Xiaoman Li, Haiyan Hu

**Affiliations:** 1 Department of Computer Science, University of Central Florida, Orlando, FL 32816 USA; 2 Nonagen BioScience Corp, Jacksonville, FL 32216 USA; 3 Department of Health Sciences Research, Mayo Clinic, Jacksonville, FL 32224 USA; 4 Burnett school of Biomedical Science, College of Medicine, University of Central Florida, Orlando, FL 32816 USA

## Abstract

Scientists have discovered various prognostic gene signatures (GSs) in different cancer types. Surprisingly, although different GSs from the same cancer type can be used to measure similar biological characteristics, often rarely is there a gene shared by different GSs. To explain such a paradox, we hypothesized that GSs from the same cancer type may be regulated by common regulatory motifs. To test this hypothesis, we carried out a comprehensive motif analysis on the prognostic GSs from five cancer types. We demonstrated that GSs from individual cancer type as well as across cancer types share regulatory motifs. We also observed that transcription factors that likely bind to these shared motifs have prognostic functions in cancers. Moreover, 75% of the predicted cofactors of these transcription factors may have cancer-related functions and some cofactors even have prognostic functions. In addition, there exist common microRNAs that regulate different GSs from individual cancer types and across cancer types, several of which are prognostic biomarkers for the corresponding cancer types. Our study suggested the existence of common regulatory mechanisms shared by GSs from individual cancer types and across cancer types, which shed light on the discovery of new prognostic GSs in cancers and the understanding of the regulatory mechanisms of cancers.

## Introduction

Studying prognostic gene signatures (GSs) can revolutionize our understanding of cancers^1^. In a given cancer type, a prognostic GS is a group of genes whose mRNA expression levels significantly associate with the tumor diagnosis, the tumor prognosis, or the therapeutic response of this cancer type^2^. Studying prognostic GSs thus greatly facilitates the understanding of tumor biology, the prediction of cancer progression, and the treatment of cancers^3^.

In 2002, the first studies of prognostic GSs were published^4, 5^. Many prognostic GSs from different cancer types were discovered thereafter. For instance, for breast cancer, scientists found the prognostic GSs such as the 70-gene GS^6^, the 76-gene GS^7^, the 21-gene GS^8^, etc. These GSs predict the breast cancer prognosis under different conditions. For instance, the 70-gene GS can predict the disease outcome in patients with 1–3 positive lymph nodes^9^ and can predict adjuvant chemotherapy response in early breast cancer^10^. The 21-gene GS has prognostic value in Asian populations with ER-positive and lymph node-negative breast cancer^11^, and can predict the response to neoadjuvant exemestane therapy in postmenopausal patients with the ER-positive breast cancer^12^.

An intriguing observation on the identified prognostic GSs in literature is that different GSs from the same cancer type seldom share genes. For instance, in breast cancer, the aforementioned 76-gene GS do not share any gene with the 21-gene GS, and only share one gene with the 70-gene GS. Although different GSs from the same cancer type rarely share genes, different GSs show similar prognostic performance in predicting the status of the cancer patients^13, 14^. We thus hypothesized that there may exist a shared regulatory mechanism by different GSs from the same cancer type, or even across different cancer types.

To test this hypothesis, we carried out a comprehensive motif analysis on the prognostic GSs from five cancer types (breast cancer, colorectal cancer, leukemia, lymphoma, and lung cancer). By de novo motifs discovery and comparison, we found that there exist shared motifs by GSs in individual cancer types as well as across cancer types. Nine of the twelve transcription factors (TFs) that likely bind to these shared motifs have reported prognostic functions in cancers. We further investigated the predicted cofactors of these TFs and found that 75% of the predicted cofactors may have prognostic or cancer-related functions. Moreover, we identified common microRNAs (miRNAs) that regulate genes in different GSs from an individual cancer type and even across cancer types. Several of these miRNAs are known prognostic biomarkers in the corresponding cancer types. Our study showed the existence of possible common regulatory mechanisms of GSs from individual cancer types and across cancer types, which shed light on the discovery of GSs in cancers and the understanding of the regulatory mechanisms of cancers.

## Material and Methods

### The collected GSs and the analysis pipeline

**Figure 1:**
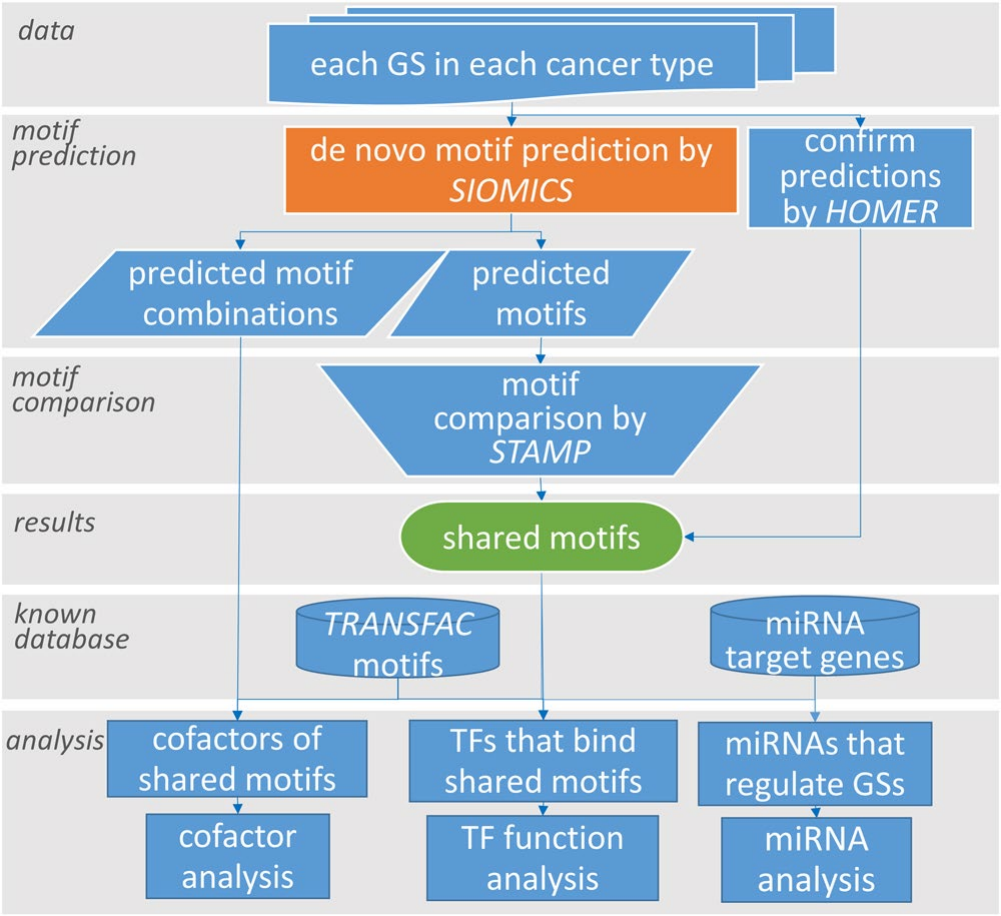
The procedure to obtain and to study the shared motifs.

We collected prognostic GSs for five cancer types: breast cancer (7 GSs), colorectal cancer (5 GSs), leukemia (6 GSs), lymphoma (6 GSs), and lung cancer (5 GSs) (Supplementary Table S1). The GSs were collected from highly cited papers by searching the keywords “prognosis” and the corresponding cancer type in Google Scholar. We only considered annotated genes at the National Center for Biotechnology Information (NCBI) in GSs in this study. We de novo predicted motifs in each GS (details in the next section). We compared the predicted motifs in different GSs from the same cancer type and across different cancer types. We observed that there exist motifs shared by GSs from an individual cancer type and even across cancer types. To corroborate the functionality of the discovered shared motifs, we studied the potential TFs that bind to these shared motifs, the cofactors of these TFs, the miRNAs that potentially regulate the GSs, etc. (Fig. 1).

### Motif discovery and comparison

For genes in each GS, we retrieved 1.5 kilobases (kb) of their upstream sequences relative to the gene translational start sites with the BioMart tool^15^ (hg38). The obtained sequences were repeat masked by RepeatMasker with the default setting (http://www.repeatmasker.org/). The de novo motifs were predicted by the SIOMICS 3.0^16, 17^ tool (http://hulab.ucf.edu/research/projects/SIOMICS/SIOMICS.html). SIOMICS can predict motifs without specifying the motif length and has been shown better performance than several popular methods^16^. In addition, it can predict cofactor motifs in the format of co-occurring motif groups, which we called motif combinations. We ran SIOMICS on the repeat-masked sequences in each GS with the default parameters except the parameter s = max (2, T*5%), where T was set as the number of sequences in a GS. This was because the default parameter s = T*1% in SIOMICS intended to work for datasets with a large number of sequences (hundreds or thousands of sequences) instead of the small number of sequences in GSs we obtained here.

We compared the predicted motifs by SIOMICS in each GS with those from all other GSs from the same cancer type. We compared motifs with their motif position weight matrices (PWMs), using the online version of STAMP^18^ with the default parameters. For each predicted motif in one GS, all motifs from other GSs with the STAMP E-value smaller than 1e-08 were considered as its similar motifs. We used the more stringent cutoff 1e-8 instead of the widely used cutoff 1e-5 in previous studies^16, 19^ to define similar motifs here, because we expected that the similarity of the predicted motifs by the same method should be high if they were the same motifs. With the defined similar motifs, we obtained all groups of similar motifs from different GSs per cancer type. We then defined shared motifs from the groups of similar motifs that contained predicted motifs from more than 50% of GSs from the same cancer type. For instance, with six GSs considered in leukemia, a shared motif in leukemia was required to be similar to at least one predicted motif in at least four GSs. For such a group of similar motifs, a shared motif was defined as the motif PWM of the motif in this group that was most similar to the known motifs in TRANSFAC^20^.

SIOMICS cannot predict motifs in small GSs well (e.g. GSs with no more than 10 genes). To corroborate the predictions by SIOMICS, and to study motifs in GSs where SIOMICS did not predict, we ran another motif discovery tool, HOMER V2^21^, with the default parameters. We randomly permuted the corresponding input sequences in each GS 20 times to obtain background sequences for HOMER. With the input sequences of genes in each GS and the corresponding background sequences, we ran HOMER with the motif length as 8, 9, 10, and 12, respectively. These motif lengths were determined by the length distribution of the motifs predicted by SIOMICS. We similarly compared these predicted motifs by HOMER with each shared motif predicted by SIOMICS.

To study the significance of the predicted motifs, we also ran SIOMICS with random sequences. For each GS, we generated random sequences by randomly permuting input sequences used by SIOMICS. That is, we randomized the order of nucleotides in input sequences to generate random sequences. We then predicted motifs in these random sequences with SIOMICS and HOMER. We repeated these steps 100 times for each GS.

### Functional analysis of the shared motifs

To obtain TFs that binds to shared motifs, we compared the shared motifs with known motifs in TRANSFAC^20^. A shared motif was claimed to be similar to a known motif if the STAMP comparison E-value was smaller than 1e-05. We used 1e-5 because the two groups of motifs compared were from different sources and we may not be able to detect similar known motifs with the above stringent cutoff. We only considered the shared motifs with similar known motifs in TRANSFAC for further analyses, because we knew their functions better. In this way, we found the TFs and their functions for about 86% of the shared motifs.

To study the function of the shared motifs, we obtained the putative target genes of a shared motif in the following way. We downloaded all 38478 human RefSeq genes (hg38) and retrieved the 1.5 kb upstream sequences of each gene from the University of California, Santa Cruz (UCSC) Genome Browser^22^ (hg38). We obtained the “target genes” of each shared motif by scanning all 38478 sequences and their reverse complements with the motif PWMs. For a motif PWM, say M = (*m*_*ij*_)_k*4_, where k is the motif length, we calculated the score of a DNA segment *s*_1_ *s*_2_ … *s*_*k*_ as follows:

$$\frac{{\sum }_{i=1}^{k}({\sum }_{j=1}^{4}{m}_{ij}{I}_{{s}_{i}=j}-{min}_{j}{m}_{ij})}{{\sum }_{i=1}^{k}({max}_{j}{m}_{ij}-{min}_{j}{m}_{ij})},$$

where ${I}_{{s}_{i}=j}$ is an indicator function with ${I}_{{s}_{i}=j}=1$ when *s*_*i*_ is the j-th type of nucleotides (in the order of “A”, “C”, “G”, and “T”), and otherwise ${I}_{{s}_{i}=j}=0$. We defined a gene as the target gene of a shared motif when there existed a score of a segment larger than 0.9 in the corresponding upstream sequence. In this study, the cutoff 0.9 approximately corresponded to at most one mismatch allowed when we compared DNA segments with the motif consensus. Recall that SIOMICS predicted motifs and motif combinations, which were groups of motifs that significantly co-occurred in input sequences. For a motif combination which contains the shared motifs, we defined its target genes as the shared target genes of all motifs in this combination. Although there may be many false positive target genes for a motif or a motif combination, the shared functions of these putative target genes may still represent the function of a shared motif (TF), under the assumption that the false positive target genes are randomly chosen.

To study the functions of target genes of motif combinations that contain the shared motifs, we obtained computationally inferred cancer-related genes sets (C4) and oncogenic cancer-related gene sets (C6) from Subramanian *et al*.^23^. In total, we had 1047 cancer-related gene sets, which contained 14196 genes with 13224 genes included in the above RefSeq gene set. We applied a binomial test to calculate the P-values of the significance of the overlap between a cancer-related gene set and the target genes of a motif combination. With a plethora of motifs combinations predicted, we could not study every motif combination. Instead, for each cancer type, we did the analyses with one randomly selected shared motif and all its corresponding motif combinations.

### The common miRNAs that regulate different GSs

To study miRNAs that bind to target genes of the shared motifs, we used miRNA target gene sets with the label of “Good mirSVR score, Conserved miRNA human” from http://www.microrna.org, as in other studies^24^. For each predicted motif in a GS that was similar to a shared motif, we calculated the significance of the overlap between the target genes of this motif in this GS and each group of miRNA target genes by the hypergeometric test. The miRNAs with the hypergeometric testing P-value smaller than 0.05 were considered as candidate miRNAs that may regulate this GS. Since multiple shared motifs were identified in a GS, we claimed that a miRNA regulates this GS if the target genes of this miRNA significantly overlap with the target genes of more than 50% of the shared motifs in this GS. In other words, for each shared motif in this GS, the overlap of its target genes with the target genes of a miRNA that regulates this GS had a hypergeometric p-value smaller than 0.05. With the miRNAs that regulate each GS, we defined common regulating miRNAs of a cancer type as the miRNAs that regulated every GS in this cancer type. For each common regulating miRNA, we studied its prognostic functions and cancer-related functions in literature.

## Results

### Prognostic GSs shared common regulatory motifs

**Figure 2:**
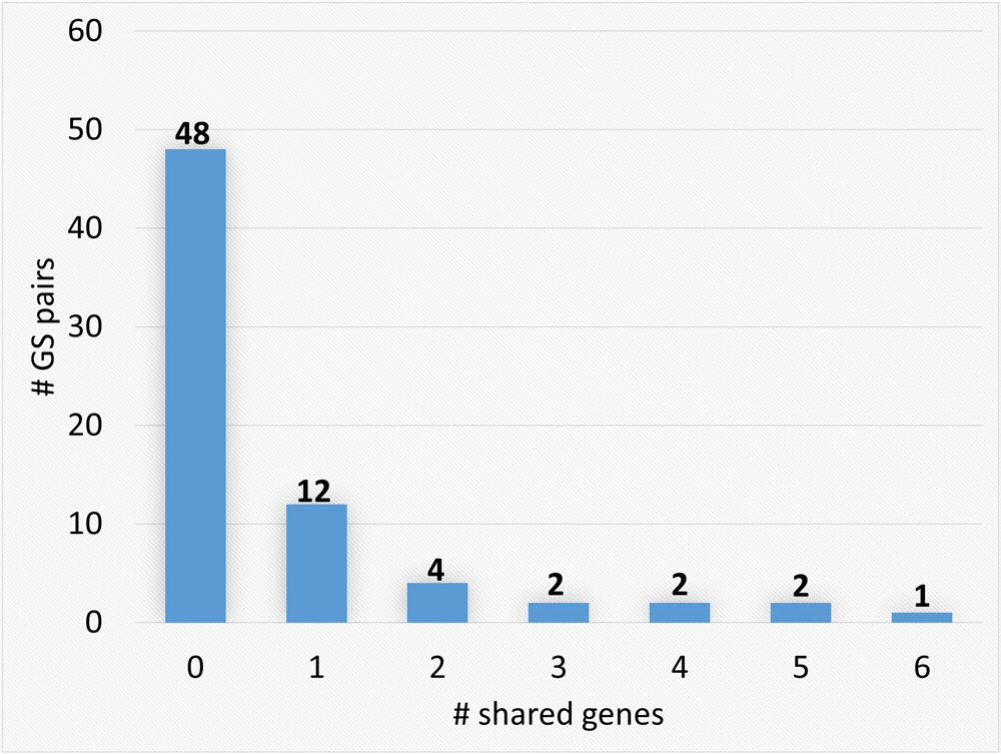
The number of genes shared by pairs of GSs from the same cancer types. All GS pairs from each cancer type were considered. The number of genes shared by GS pairs from the same cancer types is summarized in the figure.

We found that different GSs from the same cancer type seldom shared genes. We collected 7, 5, 6, 6, and 5 GSs from breast cancer, colorectal cancer, leukemia, lymphoma, and lung cancer, respectively. On average, there were 50, 57, 34, 74 and 47 genes in a GS from the corresponding cancer type, respectively (Supplementary Table S1). We found that 67% of the GS pairs from the same cancer types did not share any gene and 84% of the pairs shared at most one gene. The largest number of genes shared by two GSs from the same cancer type was only six (Fig. 2). There was no gene shared by any GS pair from different cancer types.

Since GSs from the same cancer type rarely shared genes while they characterized the same medical condition^13, 14^, we hypothesized that different GSs from the same cancer type may be regulated by common regulatory motifs. We thus applied a recently developed computational tool called SIOMICS^16, 17^ for de novo motif discovery in the proximal upstream sequences of the genes in each GS (Material and Methods).

In breast cancer, SIOMICS predicted 25, 15, 74, and 27 motifs in four of the seven GSs (GS_2, GS_4, GS_6, and GS_7). We compared these predicted motifs from different GSs by STAMP^18^ and defined similar motifs (Material and Methods). We considered a group of similar motifs from each of the four GSs as a shared motif. We represented the shared motif for a group of similar motifs by one of the similar motifs in this group that had the smallest STAMP comparison E-value when compared with known TRANSFAC motifs. Considering the predicted SIOMICS motifs, we obtained the first shared motif in breast cancer (GGGSTGGG). Similarly, we identified other six shared motifs and their corresponding TFs (Table 1).

**Table 1:** The shared motifs by GSs in each cancer type.

Cancer type	Shared motifs, their SIOMICS P-values		Most similar known motifs, TFs and the corresponding STAMP E-values	
Breast cancer	GGGSTGGG	3.88e-11	RGGSTGGG	CAC-binding protein (3.92e-11)
	GGGAGRGG	1.71e-12	GGGAGGG	MAZ (1.52e-09)
	GGGCKGGG	2.46e-12	GGGCGGGGN	SP1 (1.42e-11)
	GGCRGGGC	4.97e-10	NNGGGCGGGGCNN	GC (5.60e-10)
	CCCGGCSC	5.66e-09	NTGCACNCGGCCC	MTF1 (MTF-1) (8.63e-07)
	TGGGGCTG	7.74e-10	TNGGGGTN	KLF6 (GBF) (3.21e-08)
	GCMGCCGCC	9.65e-08	GCCGCC	BRF2 (ERF2) (1.57e-08)
Colorectal cancer	GGGSTGGG	1.96e-13	RGGSTGGG	CAC-binding protein (2.93e-11)
	CCAGCCCC	4.39e-10	CCCKCCCCN	SP1 (7.92e-08)
	GCCCCAGGCC	2.43e-09	NNNCCNCNGGCN	TFAP2A (AP-2) (4.47e-08)
	GGGGSTGG	2.99e-12	TNGGGGTN	KLF6 (GBF) (3.15e-08)
	GCCCCAGC	7.05e-08	CCGCCCNCNNC	EGR1 (KROX) (3.38e-06)
Leukemia	GGGAGRGG	2.48e-12	GGGAGGG	MAZ (1.84e-10)
	GGGCKGGG	2.55e-08	GGGCGGGGN	SP1 (4.83e-12)
Lymphoma	NGGGCGGGS	4.66e-12	NGGGGCGGGGNN	SP1 (3.83e-13)
	TGGGMGGG	4.54e-20	GGGAGGG	MAZ (3.93e-10)
	GGCKGGGC	4.48e-08	NNGGGCGGGGCNN	GC (7.99e-09)
	CKGGCTGGGG	4.39e-34	RGGSTGGG	CAC-binding protein (1.80e-06)
Lung cancer	CCCCTYCC	8.29e-21	NCCCCCCNCCC	ZNF219 (1.48e-08)
	GGKGKGRG	8.16e-09	SGGGGGGGGMNN	PATZ1 (MAZR) (4.07e-07)
	CCCGCSCC	4.25e-09	CCCKCCCCN	SP1 (3.43e-10)
	GGGTTGGGAG	8.91e-10	TTGGGAGR	IKZF1 (Lyf-1) (4.75e-08)
	AGGGCTGGGS	2.20e-12	RGGSTGGG	CAC-binding (1.51e-07)

IUPAC code is used in the motif consensus: S (G or C), R (A or G), M (A or C), K (G or T), Y (C or T), N (any base).

SIOMICS did not discover motifs in the remaining three GSs from breast cancer (Supplementary Table S2). One reason may be due to the stringent criteria used in SIOMICS, as SIOMICS requires the significant co-occurrence of a group of patterns to define motifs, which cannot be satisfied in certain datasets especially when the number of sequences from a GS is small (Supplementary Table S1). In addition, not all of the obtained GSs are of high-quality, which may prevent from identifying motifs by SIOMICS. In fact, Sanz-Pamplona *et al*. studied 31 prognostic GSs from colorectal cancer and found that only five GSs had a significant association with prognosis^25^. They also found that all GSs had low reproducibility in independent datasets. Because of the above two reasons, we did not require a shared motif occur in all GSs per cancer type. Note that we also ran another tool HOMER^21^ to predict motifs. For the three GSs which have no prediction by SIOMICS, HOMER predicted motifs similar to the shared motifs (Supplementary Table S2).

We further studied whether there existed shared motifs by GSs in other cancer types. In colorectal cancer, SIOMICS predicted five shared motifs, which were similar to the motif of the TFs SP1, the CAC-binding protein, TFAP2A (AP-2), KLF6 (GBF), and EGR1 (KROX), respectively (the names in parentheses are the corresponding TRANSFAC TF names). Note that in order to show which TRANSFAC motifs were similar to the shared motifs, we used their corresponding TRANSFAC TF names in the following analyses. In leukemia, SIOMICS discovered two shared motifs that were similar to the motifs of MAZ and SP1. In lymphoma, SIOMICS found four motifs similar to the motifs of SP1, MAZ, GC and the CAC-binding protein, respectively. In the lung cancer, SIOMICS identified five shared motifs, which were similar to motifs of ZNF219, PATZ1 (MAZR), SP1, IKZF1 (Lyf-1), and the CAC-binding protein, respectively (Table 1). HOMER predicted similar motifs in almost all GSs from each cancer type (Supplementary Table S2).

**Figure 3:**
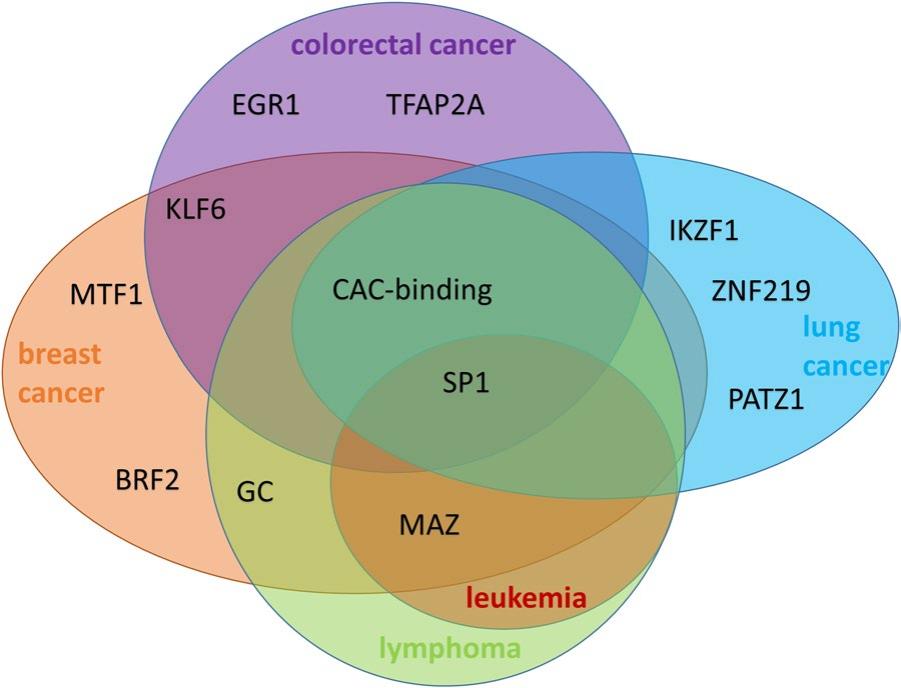
TFs of the shared motifs in each cancer type.

From Table 1, it was evident that all GSs from each cancer type shared at least two motifs. It was also obvious that GSs from different cancer types also shared the same motifs. These motifs were similar to motifs of the TFs MAZ, the CAC-binding protein, and SP1, which were discovered in at least three of the five cancer types (Fig. 3). These shared regulatory motifs indicated that not only GSs in the same cancer type but also GSs across different cancer types may share certain common regulatory mechanisms.

In addition to the significance of the shared motifs measured by their small P-values from SIOMICS (Table 1), we further investigated their significance with random sequences (Material and Methods). As expected, SIOMICS did not predict any motif in random sequences from any GS in any cancer type. For HOMER, although it predicted motifs in these random sequences, there seldom existed shared motifs among GSs per cancer type. For instance, in breast cancer, no predicted HOMER motif was shared by any three of the seven GSs. These analyses indicated that the predicted shared motifs in Table 1 were likely bona fide motifs, which also suggested that GSs from the same cancer type or different cancer types may share common regulatory mechanisms.

### TFs that bind to the shared motifs have cancer-related functions

To corroborate the functionality of the predicted shared motifs and to investigate the potential functions of these shared motifs (Table 1), we searched literature for the functions of their corresponding TFs. We found that nine of the twelve TFs that bind to the shared motifs (Table 1) have cancer-related functions in the corresponding cancer type. We also observed that at least five motifs are known prognostic biomarkers in cancers.

We found that the nine of the twelve TFs have cancer-related functions (Supplementary Table S3). For instance, SP1 and KLF6 are known tumor suppressors in colorectal cancer^26^. GC can inhibit human breast cancer cell proliferation and cancer cell-stimulated angiogenesis^27^. SP1 and MYC can modulate drug resistance of leukemia stem cells^28^. Moreover, five of the twelve TFs have been reported to be prognostic biomarkers in specific cancers, such as MAZ, KLF6 and BRF2 in breast cancer^8, 29, 30^, and ERG1 in colorectal cancer^31^. The aforementioned cancer-related functions of these TFs in their corresponding cancer types suggest that the predicted motifs are reliable and biologically significant.

Interestingly, we found that motifs shared by different GSs from the same cancer types were also shared by GSs from different cancer types (Fig. 3). For instance, motifs of the TFs SP1, MAZ, and the CAC-binding protein were shared by GSs from at least three of the five cancer types. The SP1 motif was found in all five cancer types and SP1 was found to be a prognostic biomarker in several cancer types, such as pancreatic cancer^32^, breast cancer^33^, gastric cancer^34^, lung cancer^34^, prostate cancer^35^, etc. MAZ also has cancer-related functions in three of the five cancer types^8, 36, 37^. The motif of CAC-binding protein, which was shared by four of the five cancer types, has not been well annotated yet. We expect that this motif may function in different cancer types.

### Cofactor motifs of the shared motifs have cancer-related functions

To support the functionality of the shared motifs, we investigated whether the cofactors of the TFs that bind to these shared motifs had cancer-related functions. SIOMICS output the predicted cofactor motifs of every predicted motif in motif combinations. For the twelve shared motifs, in total, there were 336 motif combinations. We obtained the cofactors of these cofactor motifs in the 336 motif combinations similarly by comparing the cofactor motifs with known motifs in TRANSFAC. We then studied the functions of the motif combinations and those of the cofactors.

First, we studied the overlap of the target genes of the 336 motif combinations with cancer-related gene sets from literature (Material and Methods). We observed that 97.6% (328/336) of the target gene sets were significantly overlapped with the cancer-related gene sets (P-value < 0.01). To assess the significance of the above overlaps, we also generated random gene groups. The number of genes in a random gene group was the same as that in the group of target genes of a corresponding motif combination. Genes in a random group were randomly selected from the 38478 RefSeq genes (Material and Methods). We found that 80.0% (269/336) of actual target gene groups have smaller P-values than their corresponding random gene groups. The mean and median of the P-values from the random groups were 0.006 and 0.008, respectively, while that from actual target gene groups were 8e-5 and 0.001, respectively. Note that it was not surprising that certain random gene groups had low P-values, because about 34.4% (13224/38478) of the RefSeq genes considered were cancer-related genes.

Second, we searched literature for the functions of each cofactor in the motif combinations that contained shared motifs. With 469 cofactor motifs in the 336 motif combinations, it was time-consuming to study each cofactor in literature. Instead, in each type of cancers, we randomly picked one shared motif and all its motif combinations to do the analysis. There were in total 44 cofactors considered in five cancer types (Supplementary Table S4). We found that about 75% (33/44) of these cofactors had cancer-related functions in literature. For instance, the silencing of the cofactor PATZ1 of KLF4 inhibits the colon cancer cell proliferation^38^. The cofactors PRDM1, EGR1, and TOPORS were shown to be tumor suppressors in colon cancer, leukemia, and lung cancer, respectively^39–41^. In addition, certain cofactors were verified to have prognostic functions in cancer. For instance, TP53 was shown to be a prognostic biomarker in breast cancer^42^. E2F was proved to be a prognostic biomarker^43^ in colorectal carcinoma. These facts corroborated the functions of the cofactor motifs and the shared motifs.

Among the 44 cofactors considered in the five cancer types, we discovered that several of them play functional roles in multiple cancers. Seven cofactors (TFAP2A, E2F, GSTM1, IL6, PATZ1, MEF2A and GTF2I) identified in two of the five cancer types have general functions in cancers. For instance, TFAP2A can regulate tumor cell migration and apoptosis^44^. Deregulated E2F activity has been found in different human cancers and correlates with poor prognosis^45^. The cofactor SPI1 occurs in three of the five cancer types. Actually, SPI1 has functions in several cancers, such as classical Hodgkin lymphoma^46^, B-cell malignancies^47^ and mixed lineage leukemia^48^. These studies in literature indicated the existence of TFs and cofactors in motif combinations, which supported the functionality of the shared motifs and the cofactor motifs in cancers.

Third, we studied whether the cofactors in the predicted motif combination indeed work together to regulate their target genes. For all twelve TFs in Table 1, cofactors of three TFs (DBP, SP1, and KLF6), coexist with their cofactors in protein complexes. For instance, in breast cancer, one of the predicted motif combinations contained both DBP and MAF. DBP and MAF together can inhibit human breast cancer cell proliferation^27^. This protein complex is also able to inhibit the growth of human pancreatic cancer in immune compromised mice^49^. In another motif combination predicted in both breast cancer and colorectal cancer, KLF6 and SP1, these two TFs together initiate the transcription of CERS2 in human prostate carcinoma cells^50^. In colorectal cancer, the predicted combination of KLF6 and E2F1 co-exist in a protein complex^51^.

Finally, we examined the cofactors of the TF that bound to the shared motifs using protein-protein interaction databases. We collected all cofactors of the seven TFs that were related to the seven shared motifs in breast cancer. Five of the seven TFs have interactive TFs in the experimentally verified TF interaction database BioGRID^52^. Among these five TFs, we discovered that two of them, SP1 and KLF6 interact with their cofactors in the motif combinations: SP1 can interact with EGR1 and TFAP2A and KLF6 can interact with SP1 and GTF3C1. In colorectal cancer, we found that the cofactors of TFAP2A were PPARG and SP1. Both PPARG and SP1 were shown to interact with TFAP2A in BioGRID. All the above evidence suggested that cofactors in the motif combinations co-exist in cancer, which supported the functions of the shared motifs.

In summary, by studying the functions of the motif combinations and those of the cofactors, we proved that TFs binding to the shared motifs may function together with their cofactors in cancers, which supported the functionality of the predicted shared motifs and their cofactor motifs.

### Common miRNAs may regulate different GSs from the same or different cancer types

Since GSs from a cancer type share common regulatory motifs, we hypothesized that these GSs may be regulated by common miRNAs as well. To test the hypothesis, we compared target genes of the shared motifs with target genes of miRNAs^53^ and identified common miRNAs that may regulate GSs (Material and Methods and Supplementary Table S5).

We predicted 61 common regulating miRNAs in breast cancer. We studied the functions of each of these miRNAs and found that about 90.2% (55/61) of them have breast cancer-related functions. For instance, hsa-mir-495 have been shown to be a novel therapeutic target for breast cancer^54^. Most importantly, we noticed that about 52.5% (32/61) of miRNAs were shown to be prognostic or potential prognostic biomarkers in breast cancer in literature (Table 2). This indicated that most of the predicted common miRNAs may have functions in prognostic GSs.

**Table 2:** The common miRNAs that were shown to be prognostic biomarkers.

Cancer type	Prognostic biomarkers
Breast cancer	hsa-mir-101, hsa-mir-106a, hsa-mir-106b, hsa-mir-129, hsa-mir-135a, hsa-mir-135b, hsa-mir-139, hsa-mir-141, hsa-mir-155, hsa-mir-15a, hsa-mir-15b, hsa-mir-181a, hsa-mir-181b, hsa-mir-181c, hsa-mir-181d, hsa-mir-185, hsa-mir-20b, hsa-mir-224, hsa-mir-27a, hsa-mir-27b, hsa-mir-30a, hsa-mir-30e, hsa-mir-339, hsa-mir-34a, hsa-mir-34c, hsa-mir-421, hsa-mir-449a, hsa-mir-494, hsa-mir-497, hsa-mir-93, hsa-mir-9, hsa-mir-96
Colorectal cancer	hsa-mir-125a, hsa-mir-125b, hsa-mir-128, hsa-mir-145, hsa-mir-15a, hsa-mir-15b, hsa-mir-16, hsa-mir-181a, hsa-mir-181b, hsa-mir-196b, hsa-mir-200c, hsa-mir-206, hsa-mir-21, hsa-mir-214, hsa-mir-218, hsa-mir-22, hsa-mir-223, hsa-mir-24, hsa-mir-339, hsa-mir-429, hsa-mir-494, hsa-mir-7, hsa-mir-708, hsa-mir-874
Leukemia	hsa-mir-106a, hsa-mir-590
Lymphoma	hsa-mir-130a, hsa-mir-135a, hsa-mir-181c, hsa-mir-200c, hsa-mir-30a, hsa-mir-34a,
Lung cancer	hsa-mir-1, hsa-mir-101, hsa-mir-124, hsa-mir-125a, hsa-mir-128, hsa-mir-130a, hsa-mir-130b, hsa-mir-146b, hsa-mir-148a, hsa-mir-148b, hsa-mir-152, hsa-mir-155, hsa-mir-17, hsa-mir-181a, hsa-mir-181b, hsa-mir-183, hsa-mir-186, hsa-mir-19a, hsa-mir-19b, hsa-mir-203, hsa-mir-204, hsa-mir-20a, hsa-mir-20b, hsa-mir-216a, hsa-mir-23a, hsa-mir-301a, hsa-mir-330, hsa-mir-33a, hsa-mir-374a, hsa-mir-377, hsa-mir-381, hsa-mir-429, hsa-mir-454, hsa-mir-494, hsa-mir-590, hsa-mir-7, hsa-mir-9, hsa-mir-93

Similar to breast cancer, we found common regulating miRNAs in other four cancer types, and most of them have cancer-related functions (Table 2). For instance, hsa-mir-181 functions as a tumor suppressor in non-small cell lung cancer^55^, hsa-mir-21 and hsa-mir-145 corporately regulate colon cancer growth and differentiation^56^. Compared with the well-studied breast cancer, we found a smaller number of prognostic biomarkers in other four cancer types (Table 2).

We noticed that 28 regulating miRNAs were shared by at least three types of cancers. For instance, two predicted common regulating miRNAs hsa-mir-106a and hsa-mir-106b, function in multiple cancer types. hsa-mir-106b expression determines the proliferation paradox of TGFB1 in breast cancer cells^57^. This miRNA also promotes colorectal cancer cell migration and invasion by directly targeting DLC1^58^. hsa-mir-106a promotes growth and metastasis of non-small cell lung cancer^59^, and its up-regulation plays an oncogenic role in pancreatic cancer^60^. In addition, hsa-mir-106a was significantly up-regulated in gastric cancer patients^61^. The above evidence showed that the identified common regulating miRNAs may have cancer-related function across different cancer types, which also suggested that the same miRNAs may regulate different GSs from the same or different cancer types.

## Discussion

We discovered multiple shared motifs by GSs from five cancer types, which implied that GSs in individual cancer types and across cancer types may be regulated by common mechanisms. We studied the functions of the potential TFs that bind to these shared motifs and the functions of the corresponding cofactors of these potential TFs and found that 75% of the TFs and cofactors may have cancer-related functions and several even have prognostic functions. Similarly, we observed that GSs from the same or different cancer types may be regulated by the same miRNAs.

SIOMICS did not find similar motifs to the shared motifs in all GSs from the same cancer type. This may be due to the quality of certain GSs used, as a previous study showed that certain prognostic GSs may be defined imperfectly^25^. This may be due to the limitation of the SIOMICS tool as well. As we pointed out above, SIOMICS cannot work well when there is no more than ten genes in a GS. This was the reason that we did not require a shared motif be shared by all GSs.

To see whether our conclusions still hold with new GSs, we further studied additional GSs for each cancer type. We predicted motifs in one additional GS for each cancer type. As expected, the majority of the aforementioned predicted shared motifs were also identified in the additional GSs (Supplementary Table S6). For instance, 71.4% (5/7) and 81.3% (13/16) of the shared motifs were discovered in the new GS from the breast cancer and from all other cancer types, respectively (Supplementary Table S6). We also noticed that three motifs (CAC-binding, MAZ and SP1) were shared by at least three of these additional GSs. This is consistent with the results from our previously collected GSs (Fig. 3). We thus concluded that although certain GSs used may prevent SIOMICS from identifying shared motifs in them, it is no doubt the GSs from the same cancer type indeed share regulatory motifs.

Scientists may define GSs more properly based on our studies. Previous studies showed that certain GSs may be defined improperly^25^. We also notice that not all GSs used in this study contain the shared motifs, which may be because that not all GSs were of the same high-quality. Based on our observation that GSs from the same cancer type may be regulated similarly, scientists may in turn define GSs better. With more widely studied GSs collected in the future, we expect that we can discover the shared motifs in an even more robust and precise way.

This study will also facilitate our understanding of the functions of TFs in cancer. About 75% of the TFs that potentially bind to the predicted shared motifs and their cofactor motifs may have cancer-related functions. Scientists can further refine prognostic TFs from the list of the predicted TFs in this study and further study the predicted common miRNAs to identify novel biomarkers in cancers.

De novo motif discovery and motif comparison are still an open problem nowadays. With better defined GSs in cancers, improved motif discovery and comparison methods, we may find more precisely shared motifs across GSs in the same or across different cancer types. In addition, similar analyses can also be carried out for other types of cancers. We anticipate that there are also exist shared regulatory mechanisms underlying these GSs.

## Electronic supplementary material

supplementary information
