# Supplementary material for: Prognostic cancer gene signatures share common regulatory motifs
Source: Sci Rep. 2017 Jul 6;7:4750. doi: 10.1038/s41598-017-05035-3 (PMC5500535; doi:10.1038/s41598-017-05035-3)
Supplement: Supplementary file 1 — supplementary information [file 41598_2017_5035_MOESM1_ESM.doc]

# Prognostic cancer gene signatures share common regulatory motifs

Ying Wang^1^, Steve Goodison^2,3^, Xiaoman Li^4*^, Haiyan Hu^1*^

^1^Department of Computer Science, University of Central Florida, Orlando, FL, 32816, USA

^2^Nonagen BioScience Corp, Jacksonville, FL, 32216, USA.

^3^Department of Health Sciences Research, Mayo Clinic, Jacksonville, FL, 32224, USA

^4^Burnett school of Biomedical Science, College of Medicine, University of Central Florida, Orlando, FL, 32816, USA

*to whom correspondence should be addressed.

Contact: [xiaoman@mail.ucf.edu](mailto:xiaoman@mail.ucf.edu), [haihu@cs.ucf.edu](mailto:haihu@cs.ucf.edu)

## Table S1 GSs collected in each cancer type.

## Table S2 shared motifs in each prognostic GS.

## Table S3 functions of shared motifs.

## Table S4 the functions of cofactors of shared motifs in motif combinations.

## Table S5 common regulating miRNAs for each cancer type

## Table S6 motifs in the additional GSs that were similar to the shared motifs.

## Table S1 GSs collected in each cancer type

| cancer type | GS_ID | #genes | genes in each GS | reference |
| --- | --- | --- | --- | --- |
| breast cancer | GS_1 | 16 | MYH4,ACOT11,SAT1,TNFSF10,PARP4,BCL2L14,ANAPC15,FUT3,CEP57,MYH2,COL2A1,RFX7,GABRQ,ZSWIM8,ZNF362,GAS2 | (Wang, et al., 2005) |
|  | GS_2 | 53 | DUSP4,EEF1A2,OR12D2,LST1,GTSE1,PSMC2,NEURL1,TMEM8A,TNFSF12,GOLM1,C3,IL18,AP2A2,MMP23B,CLN8,YIF1A,ATAD2,ABLIM1,NCAPG2,MYRF,MAP4,CD44,PHF11,FKBP2,GFOD2,SLC35A1,RRNAD1,CAPN2,CENPU,CBX3,ANKHD1,CNKSR1,FEN1,POLQ,ZFP36L2,ETV2,HIST1H4D,SMC4,ARHGDIB,TACC2,KPNA2,ORC3,NEFL,CCNE2,ACACB,UCKL1,TESPA1,ZCCHC8,PLK1,PPP1CC,BICD1,SUPT16H,TNFSF13 |  |
|  | GS_3 | 14 | E2F1,FLNC,NPTN,LOXL2,CORO1C,PTTG1,PLOD2,TAGLN,DCBLD2,MIF,MYL6,TPM2,PLAUR,FOXM1 | (Chang, et al., 2004) |
|  | GS_4 | 21 | AURKA,MYBL2,CCNB1,BCL2,CD68,GAPDH,RPLP0,CTSV,ESR1,ACTB,MMP11,SCUBE2,GUSB,GSTM1,TFRC,MKI67,GRB7,ERBB2,BIRC5,PGR,BAG1 | (Sparano and Paik, 2008) |
|  | GS_5 | 9 | MC2R,GPR27,DSTN,LCP1,RGS4,RPL3,TDRD3,HPS5,MYO1E | (Hallett, et al., 2012) |
|  | GS_6 | 181 | FDCSP,SLC25A33,C4orf32,SERTAD1,CITED4,IER5,PLP2,C9orf64,TUBB,CEBPD,ETAA1,TOB2,AIMP2,UHRF1BP1,AGPS,KLF10,FAM160B1,NUCKS1,RAD23B,RAB23,C7orf25,STK39,LYPD6B,TMEM101,ZDHHC2,KDELR3,DNMT3A,FSD1L,TMC4,MMP7,CDC123,SCNM1,NOL8,FAM53C,PGK1,CASP8,GTPBP1,CLTC,SNRPN,SWAP70,MARCH8,EIF4E2,ATIC,SMG8,DUSP10,DCAF7,CXCL2,MAST4,GOPC,GSK3B,NDEL1,CYP4V2,METTL2A,STC2,XPR1,PRSS16,CNOT4,DHRS4,NUP37,NSF,AIDA,MGP,PLAA,ELP4,DCUN1D4,BCL2,ICMT,ECHDC2,PNKD,CCNY,DPF2,BDH2,DESI2,SPIDR,PSMA5,COPB2,MLF1,PREPL,BEX5,COA7,IRF9,HSPA2,ABHD14B,INTS2,GAPDH,PEBP1,WEE1,SSR1,DNAJC13,YAE1D1,SLC38A9,ZBTB20,PDE8A,MAFF,CSNK2A1,LRPAP1,ATXN3,SLC25A25,GNPDA1,WFDC2,TMEM63A,EZR,MAPT,PAK2,SCGN,VOPP1,DNAJB1,BCDIN3D,LRP2,VTCN1,GORAB,THAP2,MAPK14,INTS8,GABARAPL1,CNIH4,DBR1,ETNK1,ERN1,PILRB,INAFM1,NAT10,RSRP1,CSTF1,SRP54,SKIV2L2,SNX6,REEP5,LDHA,ANKRD13C,STAM,SH3BGRL,EMP1,SORBS2,ARPC5,LMBR1,SLC44A1,CEP350,LTF,RNF8,ATP13A3,CD59,SPATS2L,HNMT,ALG2,SGK3,ZYG11B,GTF3C3,FLNB,SFPQ,LARS,AMMECR1,NEBL,UBE2F,MTERF2,TPD52,METTL7A,NUDT5,GOLGA8A,ERBB4,ACOT13,TYW5,FAM172A,CHPT1,TICAM2,APLP2,CIRBP,AKIP1,KLHL20,HS2ST1,ALKBH1,SNRNP25,CRACR2B,IRX3,CCDC43,THUMPD3,ETS1,AIM1,N4BP2L1,KIAA1217,ELL2 | (Liu, et al., 2007) |
|  | GS_7 | 62 | FGF18,TSPYL5,RTN4RL1,HRASLS,GPR180,MMP9,PALM2-AKAP2,NMU,DCK,CDCA7,PQLC2,ADGRG6,EGLN1,GSTM3,ZNF385B,WISP1,ECI2,RAB6A,IGFBP5,KDM7A,TMEM74B,MCM6,LPCAT1,GRHL2,ECT2,CDC42BPA,DTL,UCHL5,MS4A7,PITRM1,STK32B,SCUBE2,ALDH4A1,EXT1,CENPA,RFC4,GMPS,OXCT1,MTDH,AP2B1,BBC3,COL4A2,GNAZ,SMIM5,NDC80,SLC2A14,PRC1,FLT1,QSOX2,EBF4,CMC2,TGFB3,MSANTD3,CCNE2,DHX58,ORC6,DIAPH3,ESM1,NUSAP1,RUNDC1,FBXO31,MELK | (Weigelt, et al., 2005) |
| colorectal cancer | GS_1 | 13 | CXCL9,CXCL10,PSAT1,TLK1,MAD2L1,DLGAP5,GZMB,CA2,SLC4A4,PBK,FAS,CXCL11,TNFRSF11A | (Lin, et al., 2007) |
|  | GS_2 | 7 | ZNF148,BRI3,ELK4,ZFC3H1,ATP6V0A1,RPS5,CHD2 | (Bandrés, et al., 2007) |
|  | GS_3 | 111 | THBD,TPPP,PLA2G2E,IVL,SCN10A,DDN,CNPY3,NT5E,GPR75,EFHD2,FGL1,ADAM19,AXL,CPNE1,ANKRD40,NELFA,MRPL16,SMARCD3,URI1,PEX13,SCHIP1,HOXC4,ABTB2,DLK1,CNIH4,SLC25A10,MINA,ATN1,ASNS,CRISPLD2,BIRC5,BIN1,CNKSR1,COX7C,ASIC4,DTX4,BTG2,KLF2,CLDN16,B4GALNT1,ARL1,ASCL3,HSPA14,C4orf19,C12orf29,SCUBE2,ABCG2,ARL6IP4,PGM1,TMBIM1,SPC25,CYP2E1,PAPSS2,PCNT,NF1,C1QB,MS4A12,MRPS11,TXNIP,GTPBP2,WISP1,MS4A6A,PODNL1,EEF1G,NLK,AGGF1,BAX,PHB2,AVEN,DEDD,CTSL,CORO2B,DIP2A,ANPEP,ZSWIM8,BACH2,CASP10,SLC24A1,C12orf43,CHRDL1,MS4A1,SUPT4H1,UBE2E3,KCNQ4,NIP7,PECR,ZNF230,CDK20,DDX50,SLC39A7,TACC3,ARMC9,NOLC1,CDC14A,DECR2,SH2B1,WIPF1,TFG,COMP,CCL16,MOB3B,MADCAM1,AR,DAAM2,REEP2,NOS1,CHRNA4,CYLC1,ZSCAN32,DDX6,RPS27L | (Staub, et al., 2009) |
|  | GS_4 | 34 | CSN3,EGR1,TMEM14A,TEX11,S100A3,HPSE,DCTD,STOX2,ACTB,PRTN3,SAYSD1,NMNAT3,DENND2A,HES1,AK1,NQO1,ACKR3,MYOT,VDR,DFNB31,SPRY4,HS3ST5,SPDYA,CRABP1,MGP,CIRBP,ACYP2,MMP13,PDLIM5,SLC25A30,SYT17,MUM1L1,RALGAPA2,TACC2 | (Smith, et al., 2010) |
|  | GS_5 | 125 | SLC35D1,CEBPB,SFRP2,TNFRSF21,PRDX5,FOLR1,CTGF,PI4K2A,IRS2,GEMIN6,HTRA1,AKAP12,CCDC6,GPNMB,KCNK1,C10orf99,NDUFA4,CTSB,C4orf3,FN1,NDRG1,DUSP6,GEM,ZMPSTE24,TCHP,LSM5,EBNA1BP2,PRADC1,PXDN,SPARC,TMEM258,DYNC1H1,PPP3CA,DUSP10,FAM173B,ANXA1,CPT2,PARK7,DCBLD2,PAM,DDIT4,PODXL,ERRFI1,DLEU1,HR,APOE,WWTR1,TRIB2,BHLHE40,HIF1AN,KLF6,CD59,PLOD1,TIMP1,PAPSS1,VCAN,BGN,ALG14,KLHL8,DNAJC11,RIPK2,STX7,APIP,NADK,PLCB4,PIM1,LAMTOR4,UBE2H,VIM,SEC23IP,ZC3HC1,NNMT,ARHGEF10L,TPBG,PRKAR2A,ARHGEF40,PAFAH1B2,ALCAM,MTPAP,ZFAND5,TTC39A,P4HA1,NAPEPLD,DUSP5,NOTCH1,MCCC2,BNIP3L,CXCL16,PHLDA1,CYR61,TRIM25,COL5A2,THBS2,GADD45B,FHL2,DCAF7,LDLR,CISD3,KDSR,EARS2,RAB31,ANXA2,VCL,SPP1,BID,CD55,IGFBP3,VEGFA,R3HCC1,MYO1E,PLK2,PLAUR,C19orf43,IFI30,RBM15B,TMBIM4,SMARCE1,NDUFB7,ABHD2,MAFF,DNAJA3,MORC4,TM4SF1 | (Van Laar, 2010) |
| leukemia | GS_1 | 23 | ALS2CR8,ANGEL1,ARL6IP5,BSPRY,BTBD3,C1RL,CPT1A,DAPK1,ETFB,FGFR1,HEATR6,LAPTM4B,MAP7,NDFIP1,PBX3,PLA2G4A,PLOD3,PTP4A3,SLC25A12,SLC2A5,TMEM159,TRIM44,TRPS1,VAV3 | (Li, et al., 2013) |
|  | GS_2 | 56 | SOCS2,ABI2,ACP6,ARMCX1,ATP8B2,BCAT1,BCL11A,C10orf128,C9orf58,CD109,COL24A1,COL6A1,DAPK1,DOCK1,FAM30A,FAM92A1,FHL1,GOLGA8A,GPR56,GPSM1,GUCY1A3,HBG2,HIST1H2AD,HOPX,IL23A,IL2RA,KIAA0125,KIAA0922,LAPTM4B,LGALS3,LIMS3,LOC440995,MAP1A,MARVELD1,MAST4,MIRN155,MRC1,MSI2,MXRA7,MYO5C,NGFRAP1,NPDC1,NPL,PHGDH,PRTFDC1,RAB13,RAB34,RPL35A,RUNX1,SCD,SCHIP1,SHANK3,SLC25A37,SPARC,SYNJ2,TCF4,TESC,TM4SF1,TMEM163,TSC22D1,WBP5,ZBTB8A | (Metzeler, et al., 2008) |
|  | GS_3 | 24 | NPM1,HOXA5,HOXB6,HOXB3,PBX3,HOXB5,HOXA4,HOXB2,HOXA10,MEIS1,HOXA9,SMC4L1,PHKA2,HOXA7,ABHD2,PDGFD,NRGN,COL4A5,EMR1,HOXA6,PLEK,PRKAR2B,LTBP1,PLA2G4A,CARD9,ARHGAP22,FAM38B | (Verhaak, et al., 2005) |
|  | GS_4 | 19 | CD34,GNG7,MOX2,CCND2,ITM2C,MN1,APP,SPARC,JUP,BAALC,SMAGP,FLJ11127,SNRPN,FGFR1,PDE3B,RRAGD,CENTD1,P2RY5,MEST,MAN1A1,GPSM2,ITM2A,TRH,PGDS |  |
|  | GS_5 | 23 | LOC51334,KCNA5,GPR126,TRPS1,BHLHB3,EVI1,KIAA0977,IGHM,MMRN,MEF2C,ATP10A,FHL1,PCDHGC3,KIF17,LTBP3,PCDHGC3,SPIB,SOCS2,PCDHGC3,MEF2C,BLNK,DPP4,PROM1,SOCS2,SLC38A1,BMI1,FHL1,IRF7,SPAG6,SLC2A14,NR4A2,NR4A2,SLC2A14,NR4A2,SLC2A3,JUN,ATF3 | (Valk, et al., 2004) |
|  | GS_6 | 59 | ADFP,AGRN,AGT,AKAP2,ANGPT1,BAI2,BCL11A,CD3D,CDC42EP4,CKMT1,CNN3,COL1A1,CTNNAL1,D2S448,DAPK1,DMWD,EDG1,EML4,EMR1,FARP1,FGFR1,FHL2,FLJ21820,FLT3,FOXO1A,GLUL,GUCY1A3,GYPC,HOXA10,HOXA4,HOXB2,HOXB5,IFI27,IL6ST,ISG20,KIAA0476,KIAA0830,KIAA1447,LCN2,LOC114990,LOC55971,MAL,MAP7,MGC14376,MSI2,NBL1,NFIB,NFKB1,NFKBIB,NR2F2,NRP1,PBX3,PHEMX,PIK3R4,PLCG1,PMP22,PRG2,PRO2730,RGS16,RIS1,S100P,SCAP2,SELENBP1,SEMA3F,SFTPB,SLC2A1,SLC6A8,SLC7A7,SMG1,SNX9,SRPX,TACSTD2,TBXAS1,TCF4,TGM2,TM4SF2,TMEPAI,TUBB,UGCG,UGCGL2,VIL2 | (Radmacher, et al., 2006) |
| lymphoma | GS_1 | 32 | ASB13,HMGN1,ANKRD13A,PAG1,PTK2,MYBL1,SWAP70,NEK6,PLEKHF2,SERPINA9,ZNF608,DNMT1,STAP1,ITPKB,DNAJC10,VGLL4,MARCKSL1,FGD6,TMEM123,VNN2,TTC9,KATNAL1,KLHL5,LRMP,SSBP2,STK17A,PCYT1B,BPNT1,LMO2,MME,BCL6,VEZT | (Lenz, et al., 2008) |
|  | GS_2 | 257 | LUM,TUSC1,MFGE8,ZFPM2,PLK2,TIMP2,BGN,SMOC2,PTPRM,CHN1,MFAP2,CHI3L1,ABI3BP,C15orf48,CRIP2,TMEM173,MAFF,GSN,DCN,MRC2,EML1,DSC2,ADRA2A,LYZ,FSTL1,FAM114A1,INHBA,MICAL2,NAV2,TTYH2,PLXND1,THY1,GLT8D2,RSPO3,CTSK,FAM210B,TSC22D1,FPR3,PTPRK,ARRDC4,POSTN,CGNL1,PPP4R2,KITLG,NXN,CXCL14,HSPG2,PTTG1IP,SELM,CNN3,FN1,DZIP1,ROBO1,EDIL3,CCDC80,PKDCC,ADAMDEC1,LARP6,ANGPTL2,PKD2,PRRX1,KDELR3,TMEM47,CSRP2,ISLR,ACTA2,CSF2RA,PAPLN,GLIS2,OLFML1,APOC1,MYLK,TMEM119,ALPK2,SLC29A3,ZBED6CL,PTGDS,LAMB3,LAMB2,TNFRSF12A,PTGFRN,IL1R1,LTBP2,IFNGR1,APOE,WISP1,THBS2,UACA,THBS1,COL13A1,SULF1,PCOLCE,TGFB1I1,TMEM150C,CLU,DST,GEM,PDLIM4,SDK1,EFEMP2,RCN3,DPYSL3,TFEC,PDLIM3,CD36,VNN1,ITGB5,PMEPA1,RTN1,TSPAN6,MSRB3,ZCCHC24,VEGFC,C1orf54,SERPINF1,COX7A1,HMCN1,CAPG,FERMT2,GJA1,PMP22,TPBG,DDR2,SDC2,LRRC15,ITGAV,CYP27A1,ADGRA2,ZNF503,CNRIP1,GPR176,MMP14,ITGAX,MYO1B,TDO2,CERCAM,WLS,CTNND1,PDPN,HSPB8,SNX7,SPSB1,ITIH5,HTRA1,GAS2L1,COL16A1,GPNMB,TPM1,HBEGF,SPARC,TMEM204,CD84,OBSL1,C3,VGLL3,PARVA,DLC1,PLPP1,C10orf11,PLPP3,EVC,LAMA4,BACE1,IL7R,CSTA,COL5A2,SERPINH1,COL5A1,MXRA5,LAMP2,MXRA8,LGALS1,PRSS23,SLC27A1,YAP1,IL18,ZNF521,GPR157,WNT2,CD109,MICALL2,RARRES2,RELL1,RARRES1,CTGF,SOWAHC,EMP2,ITGB2,PARD3B,COL1A1,DNM1,LPAR1,PPIC,CTHRC1,ASAP2,SFXN3,CDH11,ANTXR1,VCAN,ADAM12,AEBP1,EDNRA,MITF,TRPA1,AFAP1,C1QTNF5,MEGF6,TEAD1,SCARA3,CEBPA,PAPSS2,GABARAPL1,RAB32,SCARA5,PLOD2,FAP,HS3ST3A1,C8orf4,ST5,SVIL,ANOS1,NHS,MPPE1,FRMD6,COL8A2,LRP12,PDGFC,CYR61,HEG1,LOXL1,COL1A2,MREG,PLAU,FBN1,PTPN21,ABCC3,HEPH,CALD1,GFPT2,CLEC11A,TAGLN,EHF,ACTN1,BNC2,IGDCC4,MCC,NRIP1,AZIN2,PTPRF,MMP9,C1S,C1R,COL6A3,COL6A2,MMP2,CHIT1,RBP5 |  |
|  | GS_3 | 61 | ITGA9,RASL12,GHR,LAPTM4B,SPARCL1,ITGA6,ROBO4,LAMB1,SPRY1,ERG,EHD2,FABP4,SORBS1,SEMA4C,CAV2,ADIPOQ,PLIN1,SAA2,PECAM1,TEK,ARAP3,ECSCR,MMRN2,LEPR,TM4SF1,TNXB,NR2F2,PALMD,AKR1C2,TFPI,RHOJ,CAV1,DLC1,KDR,ADH1B,SPON1,HOXD8,AQP1,DAAM2,ADGRF5,CYYR1,PLA2G2A,COL5A3,ADGRL4,RBP4,SAA1,IGFBP5,CXCL12,SHANK3,VWF,PRRG1,MTUS1,GULP1,GRB10,APP,CD93,SRPX,EGFL7,PTPRB,PCDH18,PROCR |  |
|  | GS_4 | 36 | IL7R,TNFSF12,CD7,HCST,TEAD1,SEMA4C,TMEM173,NOL4L,RALGDS,FNIP2,TNFRSF1B,C1RL,CCSER2,FAM46A,BIN2,SEPW1,HOXB2,PMEPA1,TNFRSF25,TTC39B,LGALS2,LEF1,CD8B,F11R,ITK,NFIC,RAB27A,ACTN1,INPP1,GALNT12,GNAQ,TNFSF13B,ATP8B2,PTRF,FLNA,ASAP2 | (Dave, et al., 2004) |
|  | GS_5 | 23 | PXDC1,ME1,FCGR1A,MITF,GPRC5B,SMG6,PELO,C4A,SEPT10,DUSP3,MRVI1,HOXD8,NDN,TLR5,OASL,HERC5,BLVRA,LGMN,F8,C3AR1,C1QB,C1QA,SCARB2 |  |
|  | GS_6 | 37 | CCL3,ZNF212,TLE1,CAT,COL3A1,SLC25A13,GCSAM,HLA-DRA,DRP2,SERPINA9,HTR2B,B4GALT1,MYC,MAP1B,PRKCB,SOD2,NR4A3,PIEZO1,HLA-DRB1,OVGP1,PLAU,CRLS1,PRKCG,PDCD4,NPM3,BMP6,ACTN1,FN1,TBP,LMO2,CCND2,CTGF,HLA-DPA1,PDE4B,BCL6,HLA-DQA1,BCL2 | (Rimsza, et al., 2008) |
| lung cancer | GS_1 | 17 | HMMR,ERBB3,DLG2,ANXA5,STAT2,STAT1,IRF4,CPEB4,ZNF264,MTOR,NF1,TBP,HGF,MMD,RNF4,DUSP6,LCK | (Wu, et al.) |
|  | GS_2 | 63 | MAP4K1,SNX1,TMSB4X,DTNA,SLC7A1,CASP10,PPOX,CCR2,LY6D,SLC2A1,ADAM17,ARHGEF1,LARS2,IKZF1,ARL4A,PEBP1,GNAT2,BIK,CXCR2,ITSN1,PSEN1,PCDHGA12,RAE1,SON,GLI2,CDH8,NID1,GOLGA1,PIK3R1,NR1H4,NTRK3,VGLL1,PYGL,TRA2A,ZNF410,SMC1A,PIGC,ENPP2,FBN2,DSP,BLM,PLEC,CHERP,STC1,PKNOX1,CRABP1,INHA,MLLT10,UPK2,ZNF154,NOTCH3,RAB28,HNRNPD,IRS1,CASP8,FUCA1,MEF2C,PFN2,PRKACA,APC,HLTF,ABCC1,BCL2 | (Lu, et al., 2006) |
|  | GS_3 | 63 | FAM198A,CD53,HSD17B11,KCNE3,TMSB4X,PABPC1,IGHA1,NQO2,SHROOM1,IGLV6-57,SLC4A3,IGKC,PARK2,FKBP9,RASL11B,ADAMTSL2,CNIH3,TRIM45,ASAH1,CD38,PLA2G7,TPD52,CD48,ADRA2C,WFDC10B,GNPTAB,EAF2,MUM1L1,FAM129A,OBSL1,CEACAM5,SULT1C2,EXT2,TRO,PSMA6,HMGCL,ERLEC1,TCEA2,CTSF,MUC4,SERP2,RHOH,ACOT8,CDK20,GIMAP7,PLEK,GSTT2,ACSS3,AOAH,C19orf57,PRDM13,TAGAP,IFI6,C1QTNF3,CLEC4E,TMEM74B,USP51,PURB,MED13L,IL11RA,IGHV3OR16-12,CYTIP,GPSM1 | (Roepman, et al., 2009) |
|  | GS_4 | 45 | STX1A,RPL27A,NACA,RPS3,ALDH1A1,SEC31A,MS4A1,PTPRCAP,GAP43,RFTN1,PDE7A,FIG4,ARHGDIB,IRX5,MAP1A,TRIO,RNASE2,DAXX,GTF2H2,AMFR,GPC3,POU5F1,ALDH9A1,IFRD2,CXCL12,P2RY6,INHA,IRF2,CXCL3,PEX7,CNN3,RPL34,MST1R,PTPN9,SLC2A1,FUCA1,MAP3K12,CORO1A,FUT3,SPOCK2,PRKACB,IGLL1,DBP,PAK1,BTK | (Sun, et al., 2008) |
|  | GS_5 | 48 | PRNP,POLG2,TRMT61B,TMEM155,DDOST,PKP3,JPH4,DENND1B,ANXA4,ZBTB11,RPS4Y1,CDC23,CPNE1,GPR183,KRT84,VIT,ZNF8,RSBN1,SIAE,MARK3,DUSP5,ZNF268,PCDHGC3,ZMYM2,ODF2,ZBTB1,FBXO32,ATXN3L,FHOD3,MAST4,ZNF827,OPA1,CLU,ZPBP,AKAP4,API5,BRAP,GULP1,CXCL8,ARMCX3,CYB5A,MGP,RPP14,HGFAC,RHBDD1,KDM5D,NFIX,PTPRH | (Larsen, et al., 2007) |

## Table S2 shared motifs in each prognostic GS

| shared motifs from SIOMICS | | | | shared motifs from HOMER | | | |
| --- | --- | --- | --- | --- | --- | --- | --- |
| cancer_ID | representative motifID_GSID | Evalue | motif from SIOMICS | cancer_ID | representative motifID_GSID | Evalue | motif from HOMER |
| Breast Cancer | | | | | | | |
| breast_2 | 43_6 | 1.69E-10 | CCCGSCCC | breast_1 | 9_7 | 8.66E-08 | TCCCAGCCA |
|  | 9_7 | 8.37E-09 | CCCAGCCT |  | 2_4 | 1.18E-07 | TCCCAGCCA |
|  | 14_6 | 1.78E-14 | CCYCTCCC |  | 14_6 | 1.21E-06 | GGGAGGAG |
|  | 13_2 | 6.55E-15 | GGCRGGGC |  | 43_6 | 1.21E-07 | GGGGCCTGG |
|  | 2_4 | 1.17E-08 | CCCAGCCT | breast_2 | 43_6 | 1.94E-06 | CCCCGCCCC |
|  | 14_2 | 6.55E-15 | TGGGGCTG |  | 14_2 | 1.78E-06 | CWGCCMTCCCCA |
| breast_4 | 43_6 | 2.81E-07 | GGCCCGGG |  | 14_6 | 1.76E-08 | GGGGAGAGA |
|  | 9_7 | 6.88E-15 | CCCASCCC |  | 13_2 | 2.31E-08 | GGGGCGGGG- |
|  | 14_6 | 6.80E-11 | CSCCTCCC |  | 2_4 | 2.56E-11 | GGGGCGGGG |
|  | 13_2 | 2.75E-08 | GGSGSGGS |  | 9_7 | 5.34E-08 | TCCCAGCCT |
|  | 2_4 | 6.55E-15 | CCCMGCCC | breast_3 | 43_6 | 1.16E-06 | CCCGCCCC |
|  | 25_7 | 8.97E-12 | GCCGCCGC- |  | 9_7 | 2.67E-11 | CCCAGSCC |
| breast_6 | 43_6 | 6.55E-15 | GSGCCGGG |  | 14_6 | 4.07E-08 | TGGGAGGG- |
|  | 9_7 | 6.55E-15 | CCCASCCC |  | 13_2 | 1.00E-09 | NGGGGCGGGGCN |
|  | 14_6 | 6.55E-15 | CCYCTCCC |  | 2_4 | 3.17E-11 | NCCNGSCC |
|  | 13_2 | 5.28E-13 | GGCGGGGC |  | 25_7 | 5.31E-09 | GGTAGCGGCTGC |
|  | 2_4 | 2.75E-11 | CNCCGCCCC | breast_4 | 43_6 | 6.62E-07 | GCTCCGGGC |
|  | 14_2 | 7.72E-09 | -GGGGCTGGCT |  | 25_7 | 4.84E-10 | -GCGGCTGCA |
|  | 25_7 | 8.88E-16 | GCCGCCGCC |  | 9_7 | 1.00E-11 | ACCCAGCCC |
| breast_7 | 43_6 | 1.22E-10 | GGGCSGGG |  | 14_6 | 4.41E-10 | GGGAGGGGGAGG |
|  | 9_7 | 6.55E-15 | CCCASCCC |  | 13_2 | 9.48E-10 | GRGGCGGGGCCA |
|  | 14_6 | 7.10E-11 | CCTCCCNC |  | 2_4 | 3.12E-12 | ACCCAGCCC |
|  | 13_2 | 8.55E-12 | GGCGGGGCCG |  | 14_2 | 1.08E-12 | CTGGGGCTGG |
|  | 2_4 | 3.15E-11 | CCSCGCCC | breast_5 | 25_7 | 8.55E-15 | GCCGCCGCC |
|  | 14_2 | 7.72E-09 | -GGGGCTGCGG |  | 9_7 | 7.84E-11 | AGGGGTGGGG |
|  | 25_7 | 1.11E-16 | GCMGCCGCC |  | 14_6 | 6.65E-07 | CYNCCCTCCC |
|  |  |  |  |  | 13_2 | 2.71E-09 | CGGCGGGG- |
|  |  |  |  |  | 2_4 | 1.17E-08 | CGGCGGGG |
|  |  |  |  |  | 14_2 | 3.60E-11 | STGGGGCTGG |
|  |  |  |  | breast_6 | 43_6 | 1.26E-06 | CCCCRCCC |
|  |  |  |  |  | 9_7 | 3.56E-11 | SCCCCACCCC |
|  |  |  |  |  | 14_6 | 3.52E-11 | TGGGGAGAGG |
|  |  |  |  |  | 13_2 | 9.64E-11 | GGGCGGGGCCTG |
|  |  |  |  |  | 2_4 | 6.58E-13 | GGGYGGGG |
|  |  |  |  |  | 25_7 | 6.66E-15 | GGCGGCGGC |
|  |  |  |  | breast_7 | 43_6 | 1.53E-06 | CCCCGCCC |
|  |  |  |  |  | 9_7 | 3.97E-11 | CCCAGCCCAG |
|  |  |  |  |  | 14_6 | 6.61E-11 | CTCCTCTCCC |
|  |  |  |  |  | 13_2 | 2.43E-12 | GGGCGGGGC |
|  |  |  |  |  | 2_4 | 6.25E-13 | GGGCGGGG |
|  |  |  |  |  | 14_2 | 4.04E-08 | CCTGCCCCA |
|  |  |  |  |  | 25_7 | 5.74E-14 | GCCGCCGCCG |
| Colorectal cancer | | | | | | | |
| colon_3 | 0_4 | 1.14E-13 | CCAGSCCC | colon_1 | 0_4 | 3.14E-09 | CCAGCCCAG |
|  | 27_3 | 0.00E+00 | GCCCCAGGCC |  | 27_3 | 2.02E-10 | -CCCCAGCCC |
|  | 2_4 | 7.72E-09 | GCCCCAGGCC |  | 2_4 | 4.03E-11 | -CCCCNGC |
|  | 10_3 | 6.55E-15 | CCCASCCC |  | 10_3 | 6.38E-11 | CCCCAGCCC |
|  | 12_5 | 1.65E-11 | GGNGGTGG |  | 12_5 | 2.06E-07 | TGGGGGAGG |
| colon_4 | 0_4 | 6.55E-15 | CCAGCCCC | colon_2 | 0_4 | 5.64E-08 | GGGGGNGGG |
|  | 27_3 | 7.72E-09 | GCCCCAGC-- |  | 27_3 | 2.42E-06 | CCCYCWGGCT |
|  | 2_4 | 6.55E-15 | GCCCCAGC |  | 10_3 | 7.24E-08 | GGGGGGGG |
|  | 12_5 | 2.15E-12 | GGGGCTGG |  | 12_5 | 8.75E-08 | GGGGGGGG |
|  | 10_3 | 2.14E-13 | CCCACCCC | colon_3 | 0_4 | 2.02E-13 | CCCAGCCCC |
| colon_5 | 0_4 | 1.69E-12 | SCAGCCCCG |  | 27_3 | 6.18E-07 | --CCCAGCCCC |
|  | 27_3 | 8.39E-12 | SCCCCGGGCC |  | 2_4 | 9.06E-12 | GCTGGGGCTGGG |
|  | 2_4 | 7.72E-09 | GCCCCCAGCC |  | 12_5 | 1.02E-11 | CCCAGCCCC |
|  | 10_3 | 2.59E-10 | -CCASCCCC |  | 10_3 | 1.48E-11 | CCCAGCCCC |
|  | 12_5 | 6.55E-15 | GGGGSTGG | colon_4 | 0_4 | 5.14E-10 | -GGGCTGGG |
|  |  |  |  |  | 27_3 | 5.13E-10 | -CCCCAGGAC |
|  |  |  |  |  | 2_4 | 7.37E-07 | GGSTGGGGNMGC |
|  |  |  |  |  | 12_5 | 4.05E-09 | CCCAGCCC- |
|  |  |  |  |  | 10_3 | 2.98E-13 | CCCAGCCC |
|  |  |  |  | colon_5 | 0_4 | 6.80E-13 | TGGGGCTGGA |
|  |  |  |  |  | 27_3 | 6.15E-09 | --CCCAGGCCTCCT |
|  |  |  |  |  | 2_4 | 6.22E-08 | AGAGGCTGGGGT |
|  |  |  |  |  | 12_5 | 1.18E-10 | TGGGGCTGGA |
|  |  |  |  |  | 10_3 | 9.35E-08 | GGGCKGGG |
| Leukemia | | | | | | | |
| leukemia_1 | 5_3 | 7.63E-08 | CCCCTCCC | leukemia_1 | 5_3 | 3.17E-07 | TCCCAGCCA |
|  | 7_6 | 3.51E-13 | CCCCTCCC |  | 7_6 | 5.22E-09 | CCCCCTYCCC |
| leukemia_2 | 5_3 | 2.53E-09 | CCCANCCC | leukemia_2 | 5_3 | 1.14E-11 | CCCCAGCCC |
|  | 7_6 | 9.67E-12 | CCCCWCCC |  | 7_6 | 2.43E-13 | CCCCTCCC |
| leukemia_3 | 5_3 | 6.55E-15 | CCCMGCCC | leukemia_3 | 5_3 | 1.27E-10 | CCCCGCCCCTGG |
|  | 7_6 | 7.13E-11 | CCCCYCCC |  | 7_6 | 2.54E-13 | CCCCTCCC |
| leukemia_4 | 5_3 | 7.83E-06 | CCCYYCCC | leukemia_4 | 5_3 | 1.17E-08 | CGCCCCCKCCCC |
|  | 7_6 | 4.87E-11 | CCCYYCCC |  | 7_6 | 3.42E-13 | GGGAGGGG |
| leukemia_5 | 5_3 | 1.02E-09 | AGCCCAGCCCAG | leukemia_5 | 5_3 | 7.29E-07 | GGGGRGCGNGGM |
|  | 7_6 | 1.21E-08 | YCCYCYCC- |  | 7_6 | 9.24E-08 | AGGGAGGGAG |
| leukemia_6 | 5_3 | 2.80E-10 | CCCCNCCC | leukemia_6 | 5_3 | 4.17E-10 | CCCAGCCCCACC |
|  | 7_6 | 6.55E-15 | CCYCTCCC |  | 7_6 | 2.34E-13 | CCCCTCCC |
| Lung cancer | | | | | | | |
| lung_1 | 6_9 | 1.13E-07 | -CCCTGCCC- | lung_1 | 2_8 | 1.45E-07 | GGGGAAGGG- |
| lung_2 | 2_8 | 6.55E-15 | GGRAGGGG |  | 14_9 | 3.50E-09 | -TCCAAACCC |
|  | 14_9 | 3.00E-09 | GGGKKGGG-- |  | 7_10 | 5.51E-08 | GGGGGTGGGG |
|  | 7_10 | 2.39E-10 | GGGKKGGG |  | 6_8 | 3.20E-06 | GGGAGGGAGGGA |
|  | 6_8 | 6.55E-15 | GGAGGMAG |  | 6_9 | 2.69E-08 | GGGGNTGG-- |
|  | 6_9 | 2.38E-10 | TTCCCAGCCC- |  | 10_8 | 1.31E-06 | GGGGNTGG |
|  | 10_8 | 6.55E-15 | CCCGCSCC |  | 33_9 | 1.41E-08 | GGGGGTGGGG |
|  | 33_9 | 1.45E-10 | CCCMMCCC | lung_2 | 2_8 | 3.00E-09 | GGGGAGGGGG |
| lung_3 | 2_8 | 6.55E-15 | GGRAGGGG |  | 14_9 | 1.82E-07 | -TCCCAGCCCM |
|  | 14_9 | 3.00E-09 | GGGKKGGG-- |  | 7_10 | 1.03E-08 | GGGGNGGGG |
|  | 7_10 | 2.39E-10 | GGGKKGGG |  | 6_8 | 1.46E-10 | GGAGGAAGTG |
|  | 6_8 | 6.55E-15 | GGAGGMAG |  | 6_9 | 5.17E-11 | GGGGCTGGG- |
|  | 6_9 | 2.38E-10 | TTCCCAGCCC- |  | 33_9 | 1.98E-09 | CCCCNCCCC |
|  | 10_8 | 6.55E-15 | CCCGCSCC |  | 10_8 | 1.25E-07 | GGGGNGGGG |
|  | 33_9 | 1.45E-10 | CCCMMCCC | lung_3 | 2_8 | 7.23E-10 | TGGGGAAGGGGA |
| lung_4 | 2_8 | 1.99E-13 | NCCCYYCC |  | 14_9 | 2.49E-07 | GRGGGTGGGGAC |
|  | 14_9 | 0.00E+00 | CTCCCAACCC |  | 7_10 | 4.05E-08 | CCCCACCCCC |
|  | 7_10 | 1.73E-11 | CCCCCMCC |  | 6_8 | 8.18E-07 | GAAGGCAGAA |
|  | 6_8 | 8.28E-09 | GNAGGAAG |  | 6_9 | 0.00E+00 | CCCCAGCCCT |
|  | 6_9 | 0.00E+00 | SCCCAGCCCT |  | 10_8 | 8.15E-07 | CCCGCCCGCC |
|  | 10_8 | 2.60E-09 | CCCKCCCC |  | 33_9 | 1.31E-08 | GGGGGTGGGG |
|  | 33_9 | 5.55E-09 | CCCCCMCC | lung_4 | 2_8 | 1.92E-09 | GGGGAGGRGG |
| lung_5 | 2_8 | 2.05E-06 | GGGCGGGG |  | 14_9 | 5.89E-08 | CTCCCACACC |
|  | 14_9 | 2.38E-10 | -CCCCAACCCC |  | 7_10 | 1.99E-09 | GGTGTGGG |
|  | 7_10 | 6.55E-15 | GGKGKGRG |  | 6_8 | 5.33E-07 | CTTCCTTC |
|  | 6_9 | 9.54E-08 | CCCCAACCCC |  | 6_9 | 1.29E-12 | --CCAGCCCT |
|  | 10_8 | 3.85E-08 | -GGGCGGGG |  | 33_9 | 6.71E-09 | CCCCACCCCC |
|  | 33_9 | 1.69E-10 | CYCMCMCC |  | 10_8 | 4.35E-06 | CCCCACCCCC |
|  |  |  |  | lung_5 | 2_8 | 2.12E-08 | CCCCKNCCCT |
|  |  |  |  |  | 14_9 | 4.35E-10 | GGGGKTGGGGGG |
|  |  |  |  |  | 7_10 | 1.21E-06 | GGGGKTGGGG |
|  |  |  |  |  | 6_8 | 9.73E-12 | CTGCCTCCC |
|  |  |  |  |  | 6_9 | 3.70E-10 | GGGGGCTGGG- |
|  |  |  |  |  | 10_8 | 2.10E-06 | GGGGGCTGGG |
|  |  |  |  |  | 33_9 | 4.24E-07 | CCCCAMCCCC |
| Lymphoma | | | | | | | |
| lymphoma_2 | 88_2 | 1.11E-16 | SCCCGCCCN | lymphoma_1 | 23_2 | 6.23E-06 | CCYYCCCTC |
|  | 14_4 | 7.84E-11 | KGGCTGGGCT |  | 88_2 | 6.76E-09 | CSNCCCCNNCCC |
|  | 23_2 | 6.55E-15 | CCCKCCCA |  | 14_4 | 6.61E-08 | TGCGGGGCG |
|  | 6_2 | 0.00E+00 | CCCCAGCCMG |  | 6_2 | 3.06E-09 | CTCCCCAGCC-- |
| lymphoma_3 | 88_2 | 7.23E-12 | -CCCRCCCC | lymphoma_2 | 88_2 | 4.37E-13 | CCCCGCCC- |
|  | 14_4 | 7.84E-11 | AGGCTGGGCC |  | 14_4 | 1.40E-08 | GGGCTGGG- |
|  | 6_2 | 1.25E-12 | CCCCAGCCC- |  | 6_2 | 1.82E-11 | CCYCNGCCC- |
|  | 23_2 | 2.04E-09 | TCCCCKCCC- |  | 23_2 | 1.04E-08 | -GGGCGGGG |
| lymphoma_4 | 88_2 | 1.98E-10 | CCSCSCCC- | lymphoma_3 | 23_2 | 1.01E-07 | ACCCTCCCT |
|  | 14_4 | 6.55E-15 | GGCKGGGC |  | 88_2 | 1.11E-09 | CCCCAGCCCC |
|  | 23_2 | 2.04E-09 | ACCCCKCCC- |  | 14_4 | 1.90E-07 | CCCCAGCCCC |
|  | 6_2 | 1.63E-11 | GCCCCAGCCC- |  | 6_2 | 1.75E-11 | CCCCAGCCCC |
| lymphoma_5 | 88_2 | 1.02E-08 | -CCCCCCCC | lymphoma_4 | 23_2 | 8.16E-08 | TCCCGCCCT |
|  | 6_2 | 7.31E-10 | CTCCAGCC-- |  | 88_2 | 3.63E-08 | TCCCGCCCT |
| lymphoma_6 | 88_2 | 2.60E-13 | CCCCGCCC- |  | 14_4 | 4.78E-11 | GGCNGGGCWGGG |
|  | 14_4 | 7.04E-09 | GGGCGGGG- |  | 6_2 | 3.16E-11 | CCCCANNCC- |
|  | 23_2 | 8.02E-09 | CCCCGCCC- | lymphoma_5 | 88_2 | 2.58E-11 | -CCCGCCCCC |
|  | 6_2 | 1.17E-07 | -GCCAGCCA- |  | 14_4 | 8.27E-09 | GAGGCGSGGCCG |
|  |  |  |  |  | 23_2 | 1.01E-07 | GGGGGCGGG |
|  |  |  |  | lymphoma_6 | 88_2 | 2.92E-09 | NCCCKCCYC |
|  |  |  |  |  | 14_4 | 2.23E-06 | -CCCCNCCC |
|  |  |  |  |  | 23_2 | 1.78E-08 | CCCCNCCC- |

## Table S3 functions of shared motifs

| cancer type | most similar TF | functions(reference) |
| --- | --- | --- |
| breast cancer | CAC-binding | NA |
|  | MAZ | MAZ [indicates poor prognosis in basal-like breast cancer](http://www.ejcancer.com/article/S0959-8049(16)30841-3/abstract)(Sparano and Paik, 2008) |
|  | SP1 | Sp1 maybe participate in the invasion and metastasis of breast cancer and is one of the valuable markers indicating poor prognosis of breast cancer.(Wang, et al., 2007) |
|  | GC (DBP) | DBP-MAF inhibited human breast cancer cell proliferation and cancer cell-stimulated angiogenesis. (Pacini, et al., 2012) |
|  | MTF-1 | MTF-1 protein levels were significantly elevated in breast cancer(Shi, et al., 2010) |
|  | GBF (KLF6) | KLF6-SV1 drives breast cancer metastasis and is associated with poor survival. (Hatami, et al., 2013) |
|  | ERF2 (BRF2) | elevated BRF2 can be independently prognostic of unfavorable survival.(Koo, et al., 2015) |
| colorectal cancer | CAC-binding | NA |
|  | SP1 | Sp1 inhibition suppressed the growth of colorectal cancer but promoted apoptosis.(Zhao, et al., 2013) |
|  | AP-2 | AP-2 associates with adenomatous polyposis coli/β-catenin and inhibits β-catenin/t-cell factor transcriptional activity in colorectal cancer cells.(Li and Dashwood, 2004) |
|  | GBF (KLF6) | KLF6 is a tumor-suppressor gene frequently inactivated in colorectal cancer.(Reeves, et al., 2004) |
|  | KROX (EGR1) | Positive expression of Egr-1 was significantly associated with age, lymphovascular invasion, lymph node and distant metastasis, tumor stage and poor survival.(Myung, et al., 2014) |
| leukemia | MAZ | the immunogenic antigens PRAME and MAZ are potential candidates for immunotherapy in acute myeloid leukemia. (Greiner, et al., 2000) |
|  | SP1 | Sp1 and c-Myc modulate drug resistance of leukemia stem cells. (Zhang, et al., 2015) |
| lymphoma | SP1 | Sp1-mediated ectopic expression of T-cell lymphoma invasion and metastasis 2 in hepatocellular carcinoma(Yen, et al., 2016) |
|  | MAZ | MAZ -related factor binding site is required for the deregulation of c-Myc expression by the immunoglobulin heavy chain gene enhancers in Burkitt's lymphoma(Hu, et al., 2002) |
|  | GC (DBP) | GalNAc-DBP complexed with oleic acid may prove effective in the integrative immunotherapy of multiple myeloma and Hodgkin’s lymphoma.(Branca, et al.) |
|  | CAC-binding | NA |
| lung cancer | ZNF219 | NA |
|  | MAZR(PATZ1) | NA |
|  | SP1 | patients with overexpression of both DNMT1 and Sp1 proteins showed poor prognosis(Lin, et al., 2010) |
|  | Lyf-1 | Ectopic Ikaros expression positively correlates with lung cancer progression (Zhang, et al., 2013) |
|  | CAC-binding | NA |

## Table S4 the functions of cofactors of shared motifs in motif combinations

| TF | function |
| --- | --- |
| breast cancer | |
| Egr | Egr-1 enhances drug resistance of breast cancer by modulating MDR1 expression in a GGPPS-independent manner. |
| MAZR (PATZ1) | PATZ1 overexpression has been described in various human malignant neoplasias, including colon, testicular and breast tumors. |
| p53 | In breast cancer, p53 mutation is associated with more aggressive disease and worse overall survival. |
| PU.1 | PU.1 (SPI1) is discovered in breast cancer too. |
| colorectal cancer | |
| MAZ | [Selenite supplementation decreases expression of MAZ in HT29 human colon adenocarcinoma cells](http://www.tandfonline.com/doi/abs/10.1080/01635589609514464) |
| GCR1 |  |
| ERF2 (BRF2) | The TFIIIB subunits, BRF1 and BRF2, are differentially expressed in cancer cell lines. |
| SMAD4 | [Smad4/DPC4 silencing and hyperactive Ras jointly disrupt transforming growth factor-β antiproliferative responses in colon cancer cells](http://www.jbc.org/content/274/47/33637.short) |
| MAZR(PATZ1) | PATZ1 is overexpressed in colon carcinomas, and its silencing inhibits colon cancer cell proliferation or increases sensitivity to apoptotic stimuli of glioma cells, suggesting an oncogenic role. |
| TFII-I (GTF2I) | SULF1 was the most overexpressed gene in different types of colon cancer, followed by GTF2i, MST1, GRN, NHSL1, and SREBF2. |
| HSF (IL6) | several therapeutics targeting the IL-6/STAT3 pathway have been developed and pose a promising strategy for the treatment of colorectal cancer |
| STAT |  |
| E2F | E2F-1 overexpression sensitizes colorectal cancer cells to camptothecin |
| AP-2alpha | Loss of AP-2alpha results in deregulation of E-cadherin and MMP-9 and an increase in tumorigenicity of colon cancer cells in vivo. |
| PU.1 |  |
| BLIMP1 (PRDM1) | PRDM1, a Tumor-Suppressor Gene, is Induced by Genkwadaphnin in Human Colon Cancer SW620 Cells. |
| MEF-2 | MEF2C was identified as a potential oncogenic transcription factor associated with Colorectal cancer |
| leukemia | |
| KROX (EGR1) | Leukemia suppressor function of Egr-1 is dependent on transforming oncogene. |
| ROM |  |
| TFII-I (GTF2I) | GTF2I-RARA is a novel fusion transcript in at(7;17) variant of acute promyelocytic leukemia with clinical resistance to retinoic acid. |
| Adf-1 |  |
| Pax-6 | Using cell-type-specific markers, the expression of Pax6 was detected in 67.0% of promyelocytic leukemia zinc finger (Plzf)-positive type A spermatogonia. |
| E2F | low E2F-1 expression and p16INK4A inactivation may serve as prognostic markers for patients with this type of lymphoma. |
| STAT5B | Novel activating *STAT5B* mutations as putative drivers of T-cell acute lymphoblastic leukemia |
| Hb (GSTM1) | GSTM1 null polymorphism is capable of causing childhood acute leukemia susceptibility. |
| lymphoma | |
| HSF | Heat-induced proteolysis of HSF causes premature deactivation of the heat shock response in Nb2 lymphoma cells |
| MEF-2 | MEF2B was the only MEF2 family member strongly implicated in lymphoma development |
| LEF1 | LEF1 is a highly specific marker for the diagnosis of chronic lymphocytic B-cell leukemia/small lymphocytic B-cell lymphoma |
| HNF1 |  |
| Pax-4 | PAX4 islets expressed higher levels of B-cell CLL/lymphoma 2 (BCL-2) |
| ANT |  |
| PU.1 | Prognostic significance of PU.1 in follicular lymphoma. |
| lung cancer | |
| NGFI-C (ERG4) | ERG4 is involved in cell proliferation of small cell lung cancer through transcriptional activation of its downstream genes. |
| ZBRK1 |  |
| GC (DBP) | Preservation of serum DBP is a significant independent factor associated with better cancer outcome in operated lung cancer patients. |
| Sp3 | Sp1 and Sp3 transcription factors are the primary determinants for activating basal transcription of the ABCG2 gene and play an important role in maintaining the side population phenotype of lung cancer cells. |
| LUN-1 (TOPORS) | expression analyses and genetic studies have implicated TOPORS as a tumor suppressor in colon, lung, brain, and prostate malignancies |
| Olf-1 |  |
| SREBP-1 |  |
| AP-2alphA | Cigarette smoke condensate decreased AP-2alpha expression by suppressing its transcription in human lung cancer cell lines, particularly in p53 deficient NCI-H1299 cells. |
| TAL1 |  |
| LF-A1 | LFA-1 was uniformly expressed in tumor-infiltrating lymphocytes from each of the nine tumors and all of the lymphocytes in non-neoplastic lung cancer type. |
| PBF |  |
| Hb (GSTM1) | Carrying the GSTM1 and GSTT1 null genotype is not a risk factor for lung cancer |

## Table S5 common regulating miRNAs for each cancer type

| cancer type | #miRNAs | shared miRNAs across different shared motifs |
| --- | --- | --- |
| lung | 73 | mir-1,mir-101,mir-106a,mir-106b,mir-124,mir-125a,mir-1271,mir-128,mir-129,mir-130a,mir-130b,mir-132,mir-139,mir-144,mir-146b,mir-148a,mir-148b,mir-152,mir-155,mir-17,mir-181a,mir-181b,mir-181c,mir-181d,mir-183,mir-185,mir-186,mir-19a,mir-19b,mir-203,mir-204,mir-20a,mir-20b,mir-211,mir-214,mir-216a,mir-218,mir-22,mir-23a,mir-23b,mir-301a,mir-301b,mir-302d,mir-30e,mir-326,mir-330,mir-33a,mir-33b,mir-340,mir-374a,mir-374b,mir-377,mir-381,mir-382,mir-429,mir-433,mir-448,mir-454,mir-485,mir-494,mir-495,mir-496,mir-506,mir-519d,mir-520c,mir-543,mir-590,mir-653,mir-7,mir-873,mir-876,mir-9,mir-93 |
| breast | 61 | mir-101,mir-106a,mir-106b,mir-129,mir-135a,mir-135b,mir-137,mir-139,mir-141,mir-149,mir-155,mir-15a,mir-15b,mir-17,mir-181a,mir-181b,mir-181c,mir-181d,mir-185,mir-186,mir-205,mir-20a,mir-20b,mir-216a,mir-218,mir-224,mir-23a,mir-23b,mir-27a,mir-27b,mir-28,mir-300,mir-30a,mir-30b,mir-30c,mir-30d,mir-30e,mir-339,mir-34a,mir-34c,mir-371,mir-377,mir-381,mir-383,mir-421,mir-448,mir-449a,mir-449b,mir-494,mir-495,mir-497,mir-519d,mir-539,mir-543,mir-544,mir-590,mir-873,mir-876,mir-9,mir-93,mir-96 |
| colon | 46 | mir-1,mir-125a,mir-125b,mir-128,mir-136,mir-145,mir-15a,mir-15b,mir-16,mir-181a,mir-181b,mir-181d,mir-196b,mir-19a,mir-19b,mir-200b,mir-200c,mir-206,mir-21,mir-214,mir-216a,mir-218,mir-22,mir-223,mir-24,mir-300,mir-326,mir-330,mir-339,mir-374a,mir-374b,mir-381,mir-410,mir-424,mir-429,mir-431,mir-485,mir-494,mir-497,mir-499,mir-544,mir-590,mir-613,mir-7,mir-708,mir-874 |
| leukemia | 14 | mir-106a,mir-106b,mir-129,mir-139,mir-17,mir-194,mir-203,mir-300,mir-374a,mir-381,mir-494,mir-519d,mir-590,mir-93 |
| lymphoma | 47 | mir-125a,mir-130a,mir-130b,mir-135a,mir-135b,mir-136,mir-148a,mir-152,mir-181c,mir-182,mir-183,mir-186,mir-19a,mir-19b,mir-200b,mir-200c,mir-211,mir-301a,mir-301b,mir-302a,mir-302b,mir-302c,mir-30a,mir-30b,mir-30c,mir-30d,mir-30e,mir-340,mir-342,mir-34a,mir-34c,mir-374a,mir-374b,mir-378,mir-410,mir-421,mir-422a,mir-429,mir-449a,mir-449b,mir-454,mir-488,mir-495,mir-539,mir-590,mir-876,mir-9 |

## Table S6 motifs in the additional GSs that were similar to the shared motifs

| cancer type (reference) | motifs similar to shared motifs | shared motifs’ corresponding TFs |
| --- | --- | --- |
| breast cancer(Nielsen, et al., 2010) | GGGSTGGG | CAC-binding |
|  | GGGAGGNG | MAZ |
|  | CCCGCSCC | SP1 |
|  | GCGGGGCC | GC |
|  | GGGCGGCGG | ERF2 |
| colorectal cancer(Nguyen, et al., 2015) | CCCTNCCC | CAC-binding |
|  | CCSCGCCC | SP1 |
|  | GCCCCGGGGC | AP-2 |
|  | GCCCCGGGGC | GBF |
|  | CCCYGCCC | KROX |
| leukemia(Ng, et al., 2016) | GAGGAGGG | MAZ |
| lymphoma(Zamani-Ahmadmahmudi, et al., 2017) | CCCGCGCC | SP1 |
|  | GGRRGGGR | MAZ |
| lung cancer(Van Laar, 2012) | GGGRGRGG | ZNF219 |
|  | CCCYCCCS | MAZR |
|  | CCCNCCCC | SP1 |
|  | CCTCCCACT | lyf-1 |
|  | CWGGGCTGGG | CAC-binding |

# References

Bandrés, E.*, et al.* A gene signature of 8 genes could identify the risk of recurrence and progression in Dukes' B colon cancer patients. *Oncology reports* 2007;17(5):1089-1094.

Branca, J.J.*, et al.* Eect of glycosylated vitamin D binding protein complexed with oleic acid on human myeloma and Hodgkin’s lymphoma cultures.

Chang, H.Y.*, et al.* Gene expression signature of fibroblast serum response predicts human cancer progression: similarities between tumors and wounds. *PLoS Biol* 2004;2(2):e7.

Dave, S.S.*, et al.* Prediction of survival in follicular lymphoma based on molecular features of tumor-infiltrating immune cells. *New England Journal of Medicine* 2004;351(21):2159-2169.

Greiner, J.*, et al.* Simultaneous expression of different immunogenic antigens in acute myeloid leukemia. *Experimental hematology* 2000;28(12):1413-1422.

Hallett, R.M.*, et al.* A gene signature for predicting outcome in patients with basal-like breast cancer. *Scientific reports* 2012;2.

Hatami, R.*, et al.* KLF6-SV1 drives breast cancer metastasis and is associated with poor survival. *Science translational medicine* 2013;5(169):169ra112-169ra112.

Hu, H.-M., Arcinas, M. and Boxer, L.M. A Myc-associated Zinc Finger Protein-related Factor Binding Site Is Required for the Deregulation of c-mycExpression by the Immunoglobulin Heavy Chain Gene Enhancers in Burkitt's Lymphoma. *Journal of Biological Chemistry* 2002;277(12):9819-9824.

Koo, J.*, et al.* Induction of proto-oncogene BRF2 in breast cancer cells by the dietary soybean isoflavone daidzein. *BMC cancer* 2015;15(1):905.

Larsen, J.E.*, et al.* Gene expression signature predicts recurrence in lung adenocarcinoma. *Clinical Cancer Research* 2007;13(10):2946-2954.

Lenz, G.*, et al.* Stromal gene signatures in large-B-cell lymphomas. *New England Journal of Medicine* 2008;359(22):2313-2323.

Li, Q. and Dashwood, R.H. Activator protein 2α associates with adenomatous polyposis coli/β-catenin and inhibits β-catenin/T-cell factor transcriptional activity in colorectal cancer cells. *Journal of Biological Chemistry* 2004;279(44):45669-45675.

Li, Z.*, et al.* Identification of a 24-gene prognostic signature that improves the European LeukemiaNet risk classification of acute myeloid leukemia: an international collaborative study. *Journal of Clinical Oncology* 2013:JCO. 2012.2044. 3184.

Lin, Y.-H.*, et al.* Multiple gene expression classifiers from different array platforms predict poor prognosis of colorectal cancer. *Clinical Cancer Research* 2007;13(2):498-507.

Liu, R.*, et al.* The prognostic role of a gene signature from tumorigenic breast-cancer cells. *New England Journal of Medicine* 2007;356(3):217-226.

Lu, Y.*, et al.* A gene expression signature predicts survival of patients with stage I non-small cell lung cancer. *PLoS Med* 2006;3(12):e467.

Metzeler, K.H.*, et al.* An 86-probe-set gene-expression signature predicts survival in cytogenetically normal acute myeloid leukemia. *Blood* 2008;112(10):4193-4201.

Myung, D.-S.*, et al.* Expression of early growth response-1 in colorectal cancer and its relation to tumor cell proliferation and apoptosis. *Oncology reports* 2014;31(2):788-794.

Ng, S.W.*, et al.* A 17-gene stemness score for rapid determination of risk in acute leukaemia. *Nature* 2016;540(7633):433-437.

Nguyen, M.N.*, et al.* CRC-113 gene expression signature for predicting prognosis in patients with colorectal cancer. *Oncotarget* 2015;6(31):31674-31692.

Nielsen, T.O.*, et al.* A comparison of PAM50 intrinsic subtyping with immunohistochemistry and clinical prognostic factors in tamoxifen-treated estrogen receptor–positive breast cancer. *Clinical Cancer Research* 2010;16(21):5222-5232.

Pacini, S.*, et al.* Effects of vitamin D-binding protein-derived macrophage-activating factor on human breast cancer cells. *Anticancer research* 2012;32(1):45-52.

Radmacher, M.D.*, et al.* Independent confirmation of a prognostic gene-expression signature in adult acute myeloid leukemia with a normal karyotype: a Cancer and Leukemia Group B study. *Blood* 2006;108(5):1677-1683.

Reeves, H.L.*, et al.* Kruppel-like factor 6 (KLF6) is a tumor-suppressor gene frequently inactivated in colorectal cancer. *Gastroenterology* 2004;126(4):1090-1103.

Rimsza, L.M.*, et al.* Gene expression predicts overall survival in paraffin-embedded tissues of diffuse large B-cell lymphoma treated with R-CHOP. *Blood* 2008;112(8):3425-3433.

Roepman, P.*, et al.* An immune response enriched 72-gene prognostic profile for early-stage non–small-cell lung cancer. *Clinical Cancer Research* 2009;15(1):284-290.

Shi, Y.*, et al.* The metal-responsive transcription factor-1 protein is elevated in human tumors. *Cancer biology & therapy* 2010;9(6):469-476.

Smith, J.J.*, et al.* Experimentally derived metastasis gene expression profile predicts recurrence and death in patients with colon cancer. *Gastroenterology* 2010;138(3):958-968.

Sparano, J.A. and Paik, S. Development of the 21-gene assay and its application in clinical practice and clinical trials. *Journal of Clinical Oncology* 2008;26(5):721-728.

Staub, E.*, et al.* An expression module of WIPF1-coexpressed genes identifies patients with favorable prognosis in three tumor types. *Journal of molecular medicine* 2009;87(6):633-644.

Sun, Z., Wigle, D.A. and Yang, P. Non-Overlapping and Non–Cell-Type–Specific Gene Expression Signatures Predict Lung Cancer Survival. *Journal of Clinical Oncology* 2008;26(6):877-883.

Valk, P.J.*, et al.* Prognostically useful gene-expression profiles in acute myeloid leukemia. *New England Journal of Medicine* 2004;350(16):1617-1628.

Van Laar, R. An online gene expression assay for determining adjuvant therapy eligibility in patients with stage 2 or 3 colon cancer. *British journal of cancer* 2010;103(12):1852-1857.

Van Laar, R.K. Genomic signatures for predicting survival and adjuvant chemotherapy benefit in patients with non-small-cell lung cancer. *BMC medical genomics* 2012;5(1):30.

Verhaak, R.G.*, et al.* Mutations in nucleophosmin (NPM1) in acute myeloid leukemia (AML): association with other gene abnormalities and previously established gene expression signatures and their favorable prognostic significance. *Blood* 2005;106(12):3747-3754.

Wang, Y.*, et al.* Gene-expression profiles to predict distant metastasis of lymph-node-negative primary breast cancer. *The Lancet* 2005;365(9460):671-679.

Weigelt, B.*, et al.* Molecular portraits and 70-gene prognosis signature are preserved throughout the metastatic process of breast cancer. *Cancer research* 2005;65(20):9155-9158.

Wu, Y.*, et al.* Validation of a 16-Gene Expression Signature in Non-Small-Cell Lung Cancers from FFPE Samples.

Yen, W.H.*, et al.* Sp1‐mediated ectopic expression of T‐cell lymphoma invasion and metastasis 2 in hepatocellular carcinoma. *Cancer medicine* 2016.

Zamani-Ahmadmahmudi, M., Aghasharif, S. and Ilbeigi, K. Prognostic efficacy of the human B-cell lymphoma prognostic genes in predicting disease-free survival (DFS) in the canine counterpart. *BMC Veterinary Research* 2017;13(1):17.

Zhang, Y.*, et al.* Sp1 and c-Myc modulate drug resistance of leukemia stem cells by regulating survivin expression through the ERK-MSK MAPK signaling pathway. *Molecular cancer* 2015;14(1):1.

Zhang, Z.*, et al.* Ectopic Ikaros expression positively correlates with lung cancer progression. *The Anatomical Record* 2013;296(6):907-913.

Zhao, Y.*, et al.* Inhibition of the transcription factor Sp1 suppresses colon cancer stem cell growth and induces apoptosis in vitro and in nude mouse xenografts. *Oncology reports* 2013;30(4):1782-1792.
